# Temporal Network of Depressive Symptoms across College Students with Distinct Depressive Trajectories during the COVID-19 Pandemic

**DOI:** 10.1155/2023/8469620

**Published:** 2023-07-15

**Authors:** Zijuan Ma, Jingbo Zhao, Huilin Chen, Yanqiang Tao, Yifan Zhang, Fang Fan

**Affiliations:** ^1^Center for Studies of Psychological Application, School of Psychology, and Guangdong Key Laboratory of Mental Health and Cognitive Science, South China Normal University, Guangzhou, China; ^2^Department of Psychology, School of Public Health, Southern Medical University, Guangzhou, China; ^3^Department of Psychiatry, University of Oxford, UK; ^4^Beijing Key Laboratory of Applied Experimental Psychology, Faculty of Psychology, Beijing Normal University, Beijing, China

## Abstract

**Background:**

There are marked differences in how individuals respond and adapt to depressive symptoms over time during the strain of public health emergencies; however, few studies have examined the interrelations between depressive symptoms in distinct depressive trajectories from the COVID-19 outbreak period to the COVID-19 control period. Therefore, this study conducted cross-lagged panel networks to investigate the temporal relationships between depressive symptoms across distinct depressive trajectories from the COVID-19 outbreak period (T1) to the COVID-19 control period (T2).

**Methods:**

A total of 35,516 young participants from the College Students' Behavior and Health Cohort during the COVID-19 pandemic were included in the current study. Depressive symptoms were self-reported using the nine-item Patient Health Questionnaire. Unique longitudinal relationships between symptoms during the COVID-19 pandemic were estimated using a cross-lagged panel network.

**Results:**

Longitudinal relationships across distinct depressive trajectories were unique during the COVID-19 pandemic. Specifically, suicidal ideation at T1 in the chronic- and delayed-dysfunction groups was most predictive of other symptoms at T2, whereas “sleep” at T1 in the recovery group and “lack of energy” at T1 in the resistance group may be strongly related to the remission of other depressive symptoms at T2.

**Conclusions:**

These exploratory findings demonstrate the directionality of relationships underlying individual symptoms in the youth and highlight suicidal ideation, sleep, and energy as potential influencers of other depressive symptoms across distinct depressive trajectories. Targeting those symptoms during the outbreak period of COVID 19 would theoretically have been beneficial in preventing and/or reducing the likelihood of spontaneous depression during the subsequent control period.

## 1. Introduction

Depression is the leading cause of ill health and disability worldwide, affecting 4.4% (320 million) of the world's population [1]. With the explosive spread of coronavirus disease 2019 (COVID-19) across the globe, one of the biggest global crises in generations, depression quickly became more common due to restriction of activities, transfer of educational mode to virtual learning, and changes in social life [2–5]. Correspondingly, recently published meta-analyses have pinpointed that the pooled prevalence of depression during COVID-19 among populations was 35% (95% confidence interval (CI): 33–38; [5]). Further, a meta-analysis conducted on college students during the COVID-19 pandemic reported the prevalence of depression to be 37% (95% CI: 32–42; [2, 3]). Notably, a systematic review of severe acute respiratory syndrome (SARS), COVID-19, and other infectious diseases also revealed that the pooled prevalence of depression (43%, 95% CI: 27–60) was significantly higher among university students than in other populations, including the general public, health workers, older adults, infected patients, infected survivors, and pregnant women [5]. Given the elevated prevalence of depression among college students, preventing and treating depression during the COVID-19 pandemic is of great significance.

Owing to the inherent unpredictability of the onset of depression, the duration of episodes, the number of episodes over a lifespan, and the pattern in which they occur are variable [6]. Indeed, evidence has suggested marked differences in how individuals respond and adapt to traumatic experiences over time [7]. For instance, Bonanno et al. [7] used latent class analysis to evaluate psychological functioning over time in 997 hospitalized survivors of the 2003 SARS epidemic in Hong Kong. They identified four latent classes: chronic dysfunction, delayed dysfunction, recovery, and resilience. In addition, a growing body of longitudinal studies has demonstrated the existence of heterogeneous time-course trajectories of depressive symptoms following traumatic experiences such as earthquakes [8] and the COVID-19 pandemic [9, 10], highlighting the significance of learning more about individuals who exhibit resilience and those who experience chronic difficulties [11]. Researchers have theorized four common trajectories as follows [7–10]: (1) chronic dysfunction, which is characterized by consistent moderate to severe depressive symptoms over time after the traumatic event; (2) delayed dysfunction, with initially no or minimal depressive symptoms followed by the postponed outbreak of moderate to severe symptoms; (3) recovery, which is defined as initial moderate to severe depressive symptoms but followed by gradual relief; and (4) resilience, which refers to the ability to maintain relatively stable levels of normal healthy functioning over time. Although the factors related to depressive trajectories among college students have been examined during the COVID-19 pandemic [9, 10], the substantial variation in individual symptoms and the associations among symptoms were not usually emphasized in previous studies. Recent network theory holds that psychiatric symptoms result from the causal interplay between symptoms, possibly involving feedback loops [12–14]. However, the intersymptom interplay across heterogeneous patterns of depressive symptoms remains relatively unclear. Therefore, it is vital to utilize less restrictive and more sophisticated analytic approaches to examine the dynamic relationships between depressive symptoms across distinct patterns.

Network analysis, based on network theory, is a novel approach to understanding the strength and nature of associations among symptoms [15], which is popular and prominent in clinical and psychiatric research domains. The network approach to psychopathology proposes that mental illnesses result from dynamic interactions between symptoms rather than a nonobservable common cause [13, 15]. Although numerous studies have been published on interacting associations between depressive symptoms over the past half-decade [16–18], they were unable to discern the direction of relationships based on undirected cross-sectional networks. The cross-lagged panel network (CLPN) was recently developed by Rhemtulla et al. and first utilized by Bernstein et al.; it can unravel the dynamic causality between symptoms over time using longitudinal data [19, 20]. Despite the fact that the CLPN has autoregressive routes, it may be confounded by stable individual differences of a trait-like or time-invariant nature [20]. However, it is well suited for identifying temporal effects between individual elements of a construct in panel data by computing within-timepoint (undirected) and between-timepoint (directed) associations [21]. Within a CLPN graph, circles represent autoregressive feedback loops, and edges depict cross-time effects; edge thickness reflects the strength of the effects, and arrows indicate the direction of prediction.

Researchers have recently evaluated the cross-lagged relationships between individual symptoms of depression among adolescents and adults [18, 22]. For instance, Rubin et al. found that “feelings of failure” were highly likely to lead to future “suicidal ideation” at least over ten months in the middle adolescent CLPN, while suicidal ideation at time 1 was eight times more likely to precede movement dysregulation at time 2 among the early adolescents [22]. However, the above studies did not provide insight into the interrelations across heterogeneous patterns of depression on a more micro level from the COVID-19 outbreak period to the COVID-19 control period. Therefore, we conducted CLPN analysis to investigate causal interactions among depressive symptoms across distinct patterns of depressive trajectories from the COVID-19 outbreak period to the COVID-19 control period.

## 2. Methods

### 2.1. Study Background

**Figure 1:**
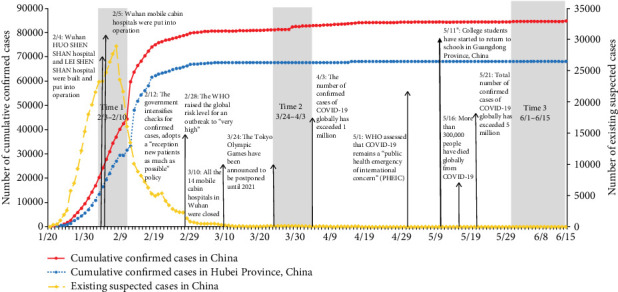
The national pandemic trend of the 2019 coronavirus disease (COVID-19) in China and sampling time windows. This figure is reproduced from Wang et al. [9, 10].

The development trend of the COVID-19 pandemic in China is shown in Figure 1. Specifically, the Chinese government announced a nationwide lockdown to control the spread of the pandemic on January 24, 2020, and we conducted the first online survey nine days after the nationwide lockdown. The total confirmed cases of COVID-19 in Mainland China increased from 17,205 to 42,638 during the first survey. During the outbreak period, the cumulative COVID-19-confirmed cases in Guangdong Province ranged from 1000 to 9999 before March 1, 2020, in a pandemic moderate-risk area as assessed by the World Health Organization in early 2020 [23]. With the effective control of the COVID-19 pandemic, China has achieved great success since April 8, 2020, and the Ministry of Education in Guangzhou Province permitted students to return to school in batches starting May 11, 2020 [2, 3]. As of 24:00 on June 1, 2020, Guangdong Province reported 1596 confirmed cases of COVID-19.

### 2.2. Participants and Procedure

**Figure 2:**
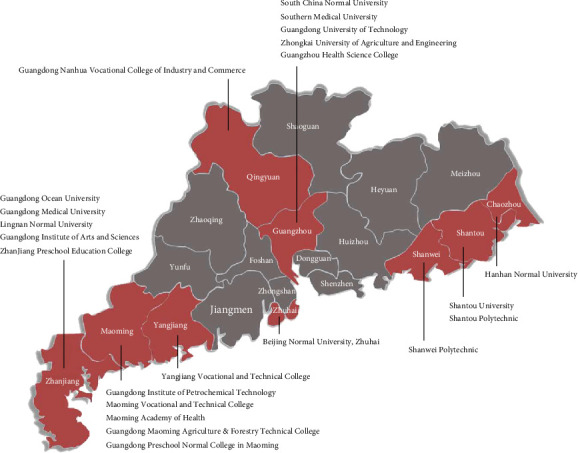
Geographical distribution of participating colleges and universities. This figure is reproduced from Zhang et al. [24].

Data were extracted from the College Students' Behavior and Health Cohort during the COVID-19 pandemic, sampling from 22 colleges/universities in Guangdong Province, China.  Figure 2 shows the geographical distribution of participating colleges and universities, and more details on sampling and data collection have been described elsewhere (Wang et al. [9, 10, 24]). During the COVID-19 pandemic, three cross-sectional online surveys were conducted from February 3 to February 10 (the outbreak period; 164,101, 88.3% valid questionnaires), March 24 to April 3 (the initial remission period; 148,343, 95.4% valid questionnaires), and June 1 to June 15 (the control period; 166,052; 88.0% valid questionnaires). After matching the three waves of data, 35,516 college students completed all three surveys and provided valid data on all measures. The current sample included two-wave surveys: the COVID-19 outbreak period (Time 1 (T1): February 3 to 10, 2020) and the COVID-19 control period (Time 2 (T2): June 1 to 15, 2020). Among the 35,516 participants, 9244 (26.0%) were male students. Detailed sample information is shown in Supplementary Table 1. The information on demographic and pandemic-related factors was collected at T1, and depressive symptoms were assessed at T1 and T2.

The current study was approved by the Human Research Ethics Committee of South China Normal University (ethics no.: SCNUPSY-2020-01-001) and carried out in line with the Helsinki Declaration, as revised in 1989. The local education bureau strongly supported our survey, and all participants or their guardians (if necessary) provided electronic informed consent before the study. In addition, they were informed of the right to withdraw from the survey at any time.

### 2.3. Measures

#### 2.3.1. Demographic and Pandemic-Related Factors

All participants completed brief demographic questions (sex and age) and pandemic-related items at T1. Pandemic-related factors included COVID-19 epidemic severity in the province of residence, confirmed or suspected cases in the community or village, relatives or friends being infected with COVID-19, and exposure to media coverage of COVID-19.

### 2.4. Depressive Symptoms

The Patient Health Questionnaire (PHQ9) was used to estimate symptoms of depression in the past two weeks at T1 and T2 [25]. Each item is rated from 0 (not at all) to 3 (nearly every day), with higher scores indicating more severe symptoms. The Chinese version of the PHQ9 has shown good psychometric properties, with Cronbach's alpha of 0.86 in the general population in China, with a recommended cut-off score of 7 or more [26, 27]. Therefore, we chose 7 as a cut-off score in this study, consistent with previous studies [9, 10, 28]. In the present study, Cronbach's alpha was 0.87 at T1 and 0.91 at T2.

### 2.5. Statistical Analyses

#### 2.5.1. Trajectories of Depressive Symptoms

We adopted two steps to generate trajectories of depressive symptoms. First, students were classified as having probable or no depressive symptoms, with a cut-off score of 7. Second, based on a previous study [9, 10], we further clustered four trajectories of depressive symptoms: chronic dysfunction, delayed dysfunction, recovery, and resistance. More specifically, individuals in the chronic-dysfunction group showed depressive symptoms at both time points. Students who reported no depressive symptoms at T1 but symptoms at T2 were assigned to the delayed-dysfunction group. Participants with depressive symptoms at T1 but without depression at T2 were included in the recovery group. For the resistance group, depressive symptoms were absent at both time points.

#### 2.5.2. Temporal Network

The R-package *glmnet* was used to construct CLPNs [29], and the R-package *qgraph* was applied to visualize all graphs of CLPNs [30, 31]. A CLPN model comprises autoregressive and cross-lagged pathways [20]. In terms of autoregressive pathways, a symptom at T1 predicts itself at T2 after controlling all other symptoms at T1. Regarding cross-lagged pathways, a symptom at T1 predicts a different symptom at T2 after adjusting for all other symptoms at T1. Moreover, to shrink small regression coefficients to 0, regression coefficients were regularized using LASSO (least absolute shrinkage and selection operator) with a 10-fold cross-validation tuning parameter selection [21].

The R-package *qgraph* was used to calculate the following centrality indices: in expected influence (in-EI) and out expected influence (out-EI) [32]. The in-EI quantifies the degree to which each symptom at T2 is predicted by other symptoms at T1 (i.e., the sum of the values of incoming edges related to a symptom), whereas the out-EI signifies the degree to which each symptom at T1 predicts other symptoms at T2 (i.e., the values of outgoing edges connected to a symptom) [18, 21, 22]. Successfully targeting a symptom with high out-EI is likely to result in the resolution of other symptoms in a longitudinal network [33, 34]. Moreover, three statistics of in-EI and out-EI are calculated: overall effects, which include all variables; cross-lagged effects, which exclude the autoregressive path of the node of interest; and cross-constructs, which exclude paths connecting nodes within the same community.

Two bootstrapping approaches implemented in the R-package *boonet* were used to measure the accuracy and stability of the CLPNs [30, 31]. First, edge weight accuracy was assessed by calculating 95% CIs around each edge weight value with nonparametric bootstrapping (1000 iterations). Second, correlation stability (CS) coefficients were evaluated using case-drop bootstrapping to determine the stability of the rank order of centrality indices. A CS value should not be less than 0.25 and preferably above 0.5 [30, 31]. Finally, edge weight difference tests were calculated to test whether edge weights differed significantly from each other.

## 3. Results

### 3.1. Depressive Trajectories

**Figure 3:**
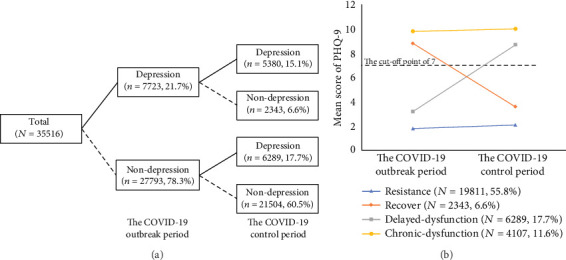
The change pattern (a) and four trajectories (b) of depressive symptoms from the COVID-19 outbreak period to the COVID-19 control period among 35,516 college students.

Figure 3 presents the change pattern and four trajectories of depressive symptoms from the COVID-19 outbreak period to the COVID-19 control period among 35,516 college students. More specifically, 5380 students (15.1%) with PHQ9 scores above the cut-off of 7 at T1 and T2 were classified in the chronic-dysfunction group. The delayed-dysfunction group, characterized by PHQ9 scores below the cut-off at T1 but equal to or above at T2, comprised 17.7% of the sample (*n* = 6289). The recovery group (*n* = 2343, 6.6%) presented an initial high level of depressive problems at T1, but no depressive symptoms at T2. Students in the resistance group (60.5%, *n* = 21,504) showed no depressive symptoms at T1 and T2.

### 3.2. Temporal Networks for Distinct Depressive Trajectories

#### 3.2.1. Network Structures

**Figure 4:**
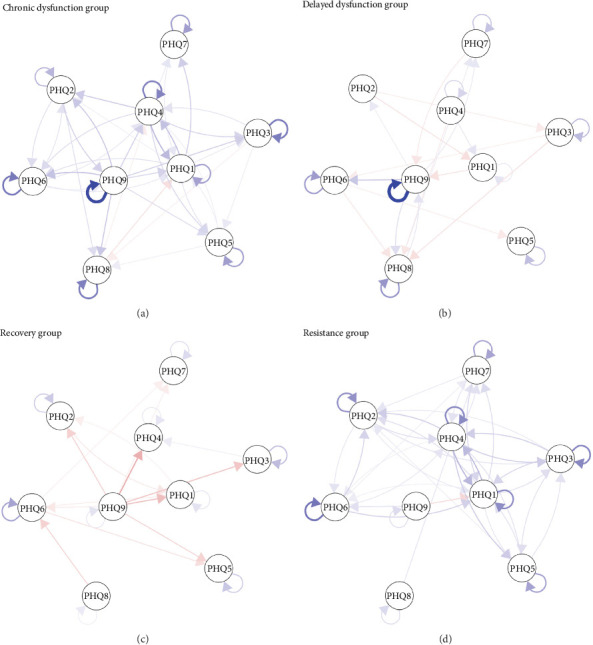
Cross-lagged panel networks (left) from the COVID-19 outbreak period to the COVID-19 control period (a: chronic-dysfunction group; b: delayed-dysfunction group; c: recovery group; d: resistance group). A threshold of all edge weights was manually set to 0.025 to make the figures more interpretable. Note: each curved arrow “loop” reflects an autoregressive association; white nodes indicate depressive symptoms and blue lines indicate positive relations, whereas orange lines signal negative relations, and line thickness and boldness reflect the strength of associations.

Figure 4 shows CLPNs from the COVID-19 outbreak period to the COVID-19 control period across four depressive trajectories. All edge weights are presented in Supplementary Tables 2–5. The CLPNs of depressive symptoms (9 nodes) revealed that 63 of 72 edges in the chronic-dysfunction group, 28 of 60 edges in the delayed-dysfunction group, 13 of 52 edges in the recovery group, and 63 of 69 edges in the resistance group were estimated to be above zero. Symptoms with the greatest autoregression coefficients were “suicidal ideation” (PHQ9) in the chronic- (edge weight = 0.345) and delayed-dysfunction groups (edge weight = 0.377) and “guilt” (PHQ6) in the recovery group (edge weight = 0.131) and the resistance group (edge weight = 0.198). Additionally, the edge “lack of energy” (PHQ4) ⟶ “anhedonia” (PHQ1) was the strongest cross-lagged edge in the chronic-dysfunction (edge weight = 0.102) and resistance groups (edge weight = 0.105). The edges “suicidal ideation” (PHQ9) ⟶ “motor” (PHQ8; edge weight = 0.049) and “suicidal ideation” (PHQ9) ⟶ “lack of energy” (PHQ4; edge weight = –0.119) displayed the strongest cross-lagged connections in the delayed-dysfunction and recovery groups, respectively.  Table 1 displays the correlation of the network structures between the four distinct trajectories. As shown in Table 1, we found correlations in the network structures between the chronic- and delayed-dysfunction groups (*r* = 0.489, *p* = 0.009) and the recovery and resistance groups (*r* = 0.413, *p* = 0.037).

#### 3.2.2. Network Inference

**Figure 5:**
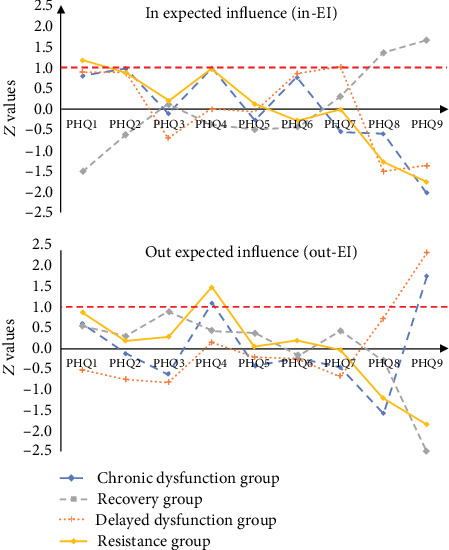
Centrality estimates of in-EI (upper) and out-EI (lower) using *z* values across four depressive trajectories. Note: centrality estimates, higher values indicate more centrality. The red line indicates the value of in-EI or out-EI equal to 1.

As shown in Figure 5 and Table 2, centrality estimates indicated that the symptoms of “depressed mood” (PHQ2; in − EI = 0.986) in the chronic-dysfunction group, “difficulty concentrating” (PHQ7; in − EI = 1.023) in the delayed-dysfunction group, “suicidal ideation” (PHQ9; in − EI = 1.668) in the recovery group, and “anhedonia” (PHQ1; in − EI = 1.185) in the resistance group had the highest in-EI. The symptoms “suicidal ideation” (PHQ9) in the chronic- (out − EI = 1.748) and delayed-dysfunction groups (out − EI = 2.328), “sleep” (PHQ3; out − EI = 0.881) in the recovery group, and “lack of energy” (PHQ4; out − EI = 1.486) in the resistance group had the highest out-EI. Given the availability and effectiveness of clinical interventions for depression [34], priority should be paid to focusing on the symptoms with high out-EI at T1 that activate other depressive symptoms at T2.

More specifically, “suicidal ideation” (PHQ9) in the chronic-dysfunction group during the COVID-19 outbreak period may have strongly motivated eight depressive symptoms during the COVID-19 control period, such as “guilt” (PHQ6; edge weight = 0.088), “depressed mood” (PHQ2; edge weight = 0.082), and “motor” (PHQ8; edge weight = 0.081). Regarding the delayed-dysfunction group, “suicidal ideation” (PHQ9) during the COVID-19 outbreak period positively predicted seven depressive symptoms during the COVID-19 control period, such as “guilt” (PHQ6; edge weight = 0.082) and “motor” (PHQ8; edge weight = 0.049). In terms of the recovery group, “sleep” (PHQ3) during the COVID-19 outbreak period positively predicted six depressive symptoms during the COVID-19 control period, such as “lack of energy” (PHQ4; edge weight = 0.027). “Lack of energy” (PHQ4) in the resistance group during the COVID-19 outbreak period positively predicted seven depressive symptoms during the COVID-19 control period, such as “anhedonia” (PHQ1; edge weight = 0.105), “appetite” (PHQ5; edge weight = 0.076), and “sleep” (PHQ3; edge weight = 0.074).

n-EI,

#### 3.2.3. Accuracy and Stability of Network Parameters

The accuracy plots of the four CLPNs show small-to-moderate confidence intervals around the edge weights, suggesting good accuracy for the baseline to follow-up networks across the four depressive trajectories (Supplementary Figure 1). Likewise, the case-drop bootstrapping results revealed that the rank order of in-EI and out-EI had moderate to strong stability across the four CLPNs (Supplementary Figure 2). Specifically, CS coefficients of in-EI and out-EI were as follows: chronic-dysfunction group: CS_in−EI_ = 0.693 and CS_out−EI_ = 0.693; delayed-dysfunction group: CS_in−EI_ = 0.493 and CS_out−EI_ = 0.336; recovery group: CS_in−EI_ = 0.321 and CS_out−EI_ = 0.679; and resistance group: CS_in−EI_ = 0.750 and CS_out−EI_ = 0.750. In addition, the edge weight difference tests (Supplementary Figure 3) revealed that these edges were significantly stronger than most other edges, and centrality difference tests (Supplementary Figures 4 and 5) indicated that these symptoms displayed significantly higher out-EI and in-EI compared with other symptoms in the CLPNs.

## 4. Discussion

To the best of our knowledge, this is the first study using CLPN analysis to investigate the progression of depressive symptoms across distinct depressive trajectories from the COVID-19 outbreak period to the COVID-19 control period among college students. We summarized four depressive trajectory groups: chronic dysfunction, delayed dysfunction, recovery, and resistance. We found that longitudinal relationships across distinct depressive trajectories were unique during the COVID-19 pandemic. Specifically, the predictive ability of “suicidal ideation” at T1 for other depressive symptoms at T2 appeared to be strongest in the chronic- and delayed-dysfunction groups, while “sleep” at T1 in the recovery group and “lack of energy” at T1 in the resistance group may be strongly related to the remission of other depressive symptoms at T2. These findings offer new insights that may help in understanding the development, maintenance, and treatment of depression.

In the present study, four depressive trajectories were identified from the COVID-19 outbreak period to the COVID-19 control period: chronic dysfunction (11.6% of the whole sample), delayed dysfunction (17.7%), recovery (6.6%), and resistance (55.8%), which is slightly inconsistent with our previously published study because data that were collected during the initial remission period of COVID-19 were excluded from the current study [9, 10]. Additionally, the proportions of chronic dysfunction and recovery in our study were lower than the rates (chronic dysfunction: 42%; recovery: 10%) estimated in a sample of hospitalized survivors of the 2003 SARS epidemic in Hong Kong, while the rates of delayed dysfunction and resistance were higher than their rates (delayed dysfunction: 13%; resistance: 35%; [7]). Notably, Bonanno [35] held that only 5% to 10% of those who are exposed to loss or potential trauma develop chronic dysfunction, which aligned with our current findings for the rate of chronic dysfunction. In addition, the epidemiological data analyzed by Bonanno et al. were collected among hospitalized survivors with extreme levels of exposure to SARS [7]; in contrast, our data were collected from college students who were in home quarantine and had a relatively low risk of being infected by COVID-19. This could explain the high rate of the resistance trajectory in our study.

“Suicidal ideation” had the highest out-EI in the chronic- (out − EI = 1.748) and delayed- (out − EI = 2.328) dysfunction groups, meaning that it most strongly predicted other depressive symptoms at T2 after adjusting for all other symptoms at T1, and demographic and pandemic-related covariates, among individuals who were depressed during the COVID-19 control period. These findings are consistent with findings from longitudinal network analysis in both early adolescence [22] and adults [18], which indicate that suicidal ideation may be particularly strongly connected with other symptoms over time. In particular, if an early adolescent reported suicidal ideation at time 1, they were eight times more likely to report movement dysregulation at time 2 [22]. Also, our findings are supported by previous cross-sectional studies in different populations [36–38]. For instance, Ballester et al. [36] suggested that prior suicidal ideation (OR = 3.17) was the strongest predictor for the persistence of depression among university students. In addition, suicidal ideation in our study may more strongly motivate symptoms of “guilt” and “motor,” which are partly consistent with other studies [22, 38]. For example, Bryan et al. [38] found that the mean level of guilt was significantly higher among military personnel with a history of suicidal ideation. The above-mentioned findings indicate that suicidal ideation could be the predictor of other depressive symptoms. This finding can be understood by the theory of suicidal trajectories and interpersonal theory of suicide, which suggest that although suicidal ideation may be strong, it is at the bottom of the pyramid; the step toward suicidal planning and attempting requires the ability to reach the peak of the pyramid (i.e., suicide; [39, 40]). Thus, most individuals who stop at the stage of suicidal thoughts might activate negative cognitions selectively, including “myself as a burden to others,” “I cannot tolerate this pain anymore,” and “I have nothing to look forward to”; these negative cognitions produce a profound sense of defeat with a sense of entrapment, which further leads to many depressive symptoms, such as “guilt” and “depressed mood” [41, 42]. This provides further evidence that suicidal ideation should be actively monitored and targeted when present. Treatments targeting suicidal ideation such as cognitive therapy for suicide prevention may provide greater reductions in suicidal ideation and prevent the co-occurrence of other depressive symptoms or decrease these symptoms [41, 43].

“Sleep” had the highest out-EI (out − EI = 0.881) estimate in the recovery group, indicating that remission in this symptom over the follow-up period was most associated with remission in all other depressive symptoms at the end of follow-up. Little is known about sleep problems and depressive symptoms in longitudinal networks; however, existing studies suggest that insomnia may increase depressed mood and other depressive symptoms and directly cause a depressive episode [44, 45]. Additionally, a meta-analysis identified insomnia as a significant predictor of the onset of depression [46]. Based on the above evidence, we speculate that the monitoring and treatment of sleep problems during the COVID-19 outbreak period, especially insomnia, may have mitigated the incidence or diminished the severity of depression during the COVID-19 control period. Cognitive behavioral therapy for insomnia, the most widely used and widely studied nondrug treatment for insomnia, should be used to intervene and prevent sleep problems during a virus outbreak period to reduce the possibility of depression developing over time [47].

“Lack of energy,” with the highest out-EI (out − EI = 1.486) in the resistance group, predicted other depressive symptoms at follow-up, which is consistent with the findings from cross-sectional network analyses in Hong Kong residents during the COVID-19 pandemic [48]. Although there is a lack of evidence from the longitudinal network, the above finding implies that improvement in “lack of energy” over time was associated with subsequent improvement in most other depressive symptoms during the COVID-19 pandemic. “Lack of energy” refers to the exhausting nature of the condition and its associated stress states [49], which were caused by the COVID-19 outbreak and its confinement measures, including contact restrictions, home quarantine, and closure of schools, colleges, and universities. In addition, “lack of energy” is closely associated with lack of motivation and cognitive dysfunction [50], and severe cognitive dysfunction (i.e., negative evaluative bias) will support the persistence of depressed mood and other depressive symptoms [42]. Therefore, exercise therapy should be implemented to enhance energy to disrupt indirectly the persistence of depressed mood [51].

Over the past decade, the application of the network approach in the study of psychopathology has developed increased popularity and prominence [52]. It holds that mental disorders result from interactions between the symptoms of the specific mental illness [12–15]. Despite calls to investigate mental illnesses at the symptom level rather than the disorder level, most investigations on this topic have been cross-sectional [16, 48], precluding study of the dynamic interplay of depressive symptoms. Our study employed the CLPN model to corroborate work on new developments in the temporal associations between symptoms hypothesized by network theory. Compared to previous CLPN networks [18, 22], advancements in our study have led to the development of temporal sequences and changes in depressive symptoms over time across distinct depressive trajectories. In addition, similar to the finding that suicide ideation was directly related to most depressive symptoms [16], our study further found that suicidal ideation was a key symptom in both the chronic- and delayed-dysfunction groups and, therefore, was common to distinct depressive trajectories. These findings indicate that future researchers might apply CLPN models to heterogeneous groups to provide more detailed information regarding dynamic effects on psychological or mental symptoms.

Clinically, identifying which interventions directly target which symptoms in which populations may be useful. Our findings have provided information on which symptoms in which populations were important during the COVID-19 pandemic by identifying the key symptoms in networks of distinct depressive trajectories over time. Specifically, “suicidal ideation” had the strongest value in predicting other depressive symptoms in the chronic- and delayed-dysfunction groups; in contrast, “sleep” in the recovery group and “lack of energy” in the resistance group were highly associated with the remission of other depressive symptoms. Hence, brief interventions targeting “sleep” and “lack of energy” may promote the spontaneous remission of depressive symptoms in the acute phase, while treatments targeting “suicidal ideation” may help minimize the subsequent development of other depressive symptoms in individuals with chronic and delayed dysfunction. Overall, in order to achieve clinically meaningful predictions, future network models should be aimed at examining the network structure of subgroups rather than focusing on the whole sample only.

Our findings should be interpreted with consideration of several limitations. First, sampling bias should be noted in our study and may be reflected in several aspects as follows: (1) All participants were recruited from colleges/universities, which limits the generalizability of our findings to other populations, including COVID-19 patients, healthcare workers, medical professionals, and older populations. (2) The surveys were conducted in Guangdong Province, which is one of the richest parts of China. Therefore, there may be bias in our findings since the resources available in response to COVID-19 varied across China. (3) Our study was similar to previous studies in that female students may be more willing to participate in mental health surveys [53–56], but a high proportion of females might affect estimates of depression. Second, self-reported depressive symptoms may be prone to reporting bias, which could hinder symptom-level analysis, particularly if an individual cannot precisely distinguish a given depressive symptom out of the many. In addition, although a growing body of longitudinal studies has indicated that most people's long-term psychological reactions can be reliably captured by four prototypical outcome patterns or trajectories (chronic dysfunction, delayed dysfunction, recovery, and resilience) across time [7, 57, 58], response bias may have resulted from simply using the sum score of depressive symptoms to divide students into four subgroups without a clinical diagnosis. Third, although we included several demographic characteristics (sex and age) and pandemic-related factors as covariates in the CLPN model to mitigate confounding effects from these sources, we did not consider other variables (e.g., history of mental illness and psychiatric drug usage) that might differentially influence or moderate the temporal interrelationships of depressive symptoms, and we did not collect any COVID-19-related information in T2. Fourth, although de Ron et al. [59] suggested that Berkson's bias is a considerable and underappreciated problem when applying the Gaussian Graphical Model and the Ising Model to clinical populations [59], there have been few studies to investigate the effect of Berkson's bias on the performance of the CLPN in clinical populations and other populations. To pay more attention to Berkson's bias, future studies should focus explicitly on the impact of Berkson's bias on other network models (i.e., the CLPN model) in different populations. Fifth, similar to other CLPN networks [21, 22, 33, 60, 61], our study also found that each item of the PHQ9 was not normally distributed according to the Kolmogorov–Smirnov test (see Supplementary Table 6). Unfortunately, we did not find an approach to estimate high dimensional directed graphical models efficiently and robustly. Future studies could pay attention to the nonparametric correlation in CLPN models. Finally, this study provides insight into predictive and potentially causal interplay among depressive symptoms across a four-month lag; however, it is still unclear what time lags are appropriate or optimal to capture relationships between individual symptoms [21]. Therefore, future research should explore various expected time lags when deciding sampling frequency.

## 5. Conclusion

With panel data from a large and population-based cohort of youths, our study applied the novel methodology of CLPN to identify unique longitudinal relationships between depressive symptoms across groups with distinct depressive trajectories over time, overcoming the limitations of past studies that failed to explore the dynamic effects of depressive symptoms during the COVD-19 pandemic. Our findings indicated that targeting symptoms of “sleep” and/or “lack of energy” during the outbreak period of public health emergencies, such as COVID 19, would theoretically be beneficial in preventing and/or reducing the likelihood of spontaneous depression during the subsequent control period. More importantly, the disappearance of “suicidal ideation” would theoretically have been accompanied by lower overall connectivity in the depressive network during the COVID-19 control period, thus promoting the rehabilitation of depressed individuals. Hence, individualized intervention was needed for young people who reported suicidal ideation during the COVID-19 outbreak.

## Figures and Tables

**Table 1:** Correlation of the network structures between groups of distinct trajectories.

		1	2	3	4
1	Chronic-dysfunction group	1			
2	Delayed-dysfunction group	0.489⁣^∗∗^	1		
3	Recovery group	-0.199	-0.201	1	
4	Resistance group	0.249	0.038	0.413⁣^∗^	1

Note: ⁣^∗^*P* < 0.05; ⁣^∗∗^*P* < 0.01.

**Table 2:** Centrality estimates of in-EI and out-EI using *z* values across four depressive trajectories.

Symptoms		Chronic-dysfunction group		Delayed-dysfunction group		Recovery group		Resistance group	
		In-EI	Out-EI	In-EI	Out-EI	In-EI	Out-EI	In-EI	Out-EI
PHQ1	Anhedonia	0.802	0.605	0.889	-0.525	-1.513	0.539	1.185	0.883
PHQ2	Depressed mood	0.986	-0.122	0.875	-0.750	-0.614	0.296	0.880	0.184
PHQ3	Sleep	-0.114	-0.615	-0.703	-0.808	0.116	0.881	0.196	0.286
PHQ4	Lack of energy	0.979	1.113	-0.002	0.145	-0.369	0.437	0.964	1.486
PHQ5	Appetite	-0.257	-0.435	-0.069	-0.219	-0.500	0.375	0.119	0.043
PHQ6	Guilt	0.775	-0.253	0.864	-0.245	-0.452	-0.172	-0.279	0.194
PHQ7	Difficulty in concentration	-0.546	-0.467	1.023	-0.655	0.303	0.429	-0.018	-0.027
PHQ8	Motor	-0.604	-1.573	-1.507	0.730	1.362	-0.292	-1.281	-1.207
PHQ9	Suicidal ideation	-2.021	1.748	-1.372	2.328	1.668	-2.493	-1.766	-1.844

Note: in-EI: in expected influence; out-EI: out expected influence.

## Data Availability

The data that support the findings of this study are available from the corresponding author upon reasonable request.

## Supplementary Materials

Supplementary Materials Detailed sample information is shown in Supplementary Table 1. All edge weights are presented in Supplementary Tables 2–5. Supplementary Figures 1–5 provide the results for accuracy (Supplementary Figure 1) and stability (Supplementary Figure 1) of the network, the edge weight difference tests (Supplementary Figure 3), and centrality difference tests (Supplementary Figures 4 and 5).
