# Supplementary material for: Temporal Network of Depressive Symptoms across College Students with Distinct Depressive Trajectories during the COVID-19 Pandemic
Source: Depress Anxiety. 2023 Jul 15;2023:8469620. doi: 10.1155/2023/8469620 (PMC11921855; doi:10.1155/2023/8469620)
Supplement: Supplementary Materials — Detailed sample information is shown in Supplementary Table 1. All edge weights are presented in Supplementary Tables 2–5. Supplementary Figures 1–5 provide the results for accuracy (Supplementary Figure 1) and stability (Supplementary Figure 1) of the network, the edge weight difference tests (Supplementary Figure 3), and centrality difference tests (Supplementary Figures 4 and 5). [file 8469620.f1.zip › Supplementary Material (1).docx]

**Supplementary Material**

Among 35,516 participants, 9,244 were male students. Detailed sample information is shown in Supplement Table 1.

**Supplementary Table 1.** Demographic and pandemic-related factors (N = 35,516)

| **Variable** | |  | ***n*** | **%** |
| --- | --- | --- | --- | --- |
| **Sex** | |  |  |  |
|  | Male |  | 9244 | 26.0 |
|  | Female |  | 26272 | 74.0 |
| **Age** | |  |  |  |
|  | Below or equal to 18 years |  | 4726 | 13.3 |
|  | Between 19 and 20 years |  | 16749 | 47.2 |
|  | Between 21 and 22 years |  | 10458 | 29.4 |
|  | Between 23 and 24 years |  | 2718 | 7.7 |
|  | Equal to or above 25 years |  | 865 | 2.4 |
| **COVID-19 epidemic severity in the living province** | |  |  |  |
|  | Mild |  | 3649 | 10.3 |
|  | Moderate |  | 31597 | 89.0 |
|  | Severe |  | 270 | 0.8 |
| **Infected cases in the community or village** | |  |  |  |
|  | No |  | 2352 | 6.6 |
|  | Yes |  | 33164 | 93.4 |
| **Relatives or acquaintances being infected with COVID-19** | |  |  |  |
|  | Nobody |  | 22250 | 62.6 |
|  | Don't know |  | 12866 | 36.2 |
|  | Confirmed or suspected |  | 400 | 1.1 |
| **Exposure to media coverage of the COVID-19** | |  |  |  |
|  | < 1 hours/day |  | 13362 | 37.6 |
|  | 1-2 hours/day |  | 16562 | 46.6 |
|  | ≥3 hours/day |  | 5592 | 15.7 |

All edge weights are presented in Supplement Table 1 - 4.

**Supplementary Table 2.** Weighted adjacency matrix from the COVID-19 outbreak period to the COVID-19 control period in the chronic dysfunction group.

|  | PHQ1 | PHQ2 | PHQ3 | PHQ4 | PHQ5 | PHQ6 | PHQ7 | PHQ8 | PHQ9 |
| --- | --- | --- | --- | --- | --- | --- | --- | --- | --- |
| PHQ1: Anhedonia | 0.136 | 0.027 | 0.035 | 0.090 | 0.043 | 0.049 | 0.073 | 0.019 | -0.017 |
| PHQ2: Depressed mood | 0.021 | 0.105 | 0.000 | 0.000 | 0.000 | 0.049 | 0.000 | 0.050 | 0.067 |
| PHQ3: Sleep | 0.009 | 0.016 | 0.186 | 0.042 | 0.032 | 0.000 | 0.025 | -0.027 | 0.000 |
| PHQ4: Lack of energy | 0.102 | 0.076 | 0.061 | 0.179 | 0.060 | 0.053 | 0.036 | 0.022 | 0.000 |
| PHQ5: Appetite | 0.029 | 0.009 | 0.012 | 0.029 | 0.140 | 0.000 | 0.018 | 0.027 | 0.005 |
| PHQ6: Guilt | 0.037 | 0.042 | 0.019 | 0.038 | 0.003 | 0.192 | 0.012 | -0.016 | 0.027 |
| PHQ7: Difficulty concentration | 0.036 | 0.007 | 0.013 | 0.019 | 0.010 | 0.020 | 0.141 | 0.021 | -0.003 |
| PHQ8: Motor | -0.048 | 0.002 | -0.008 | -0.029 | -0.007 | -0.009 | 0.000 | 0.185 | 0.024 |
| PHQ9: Suicidal ideation | 0.064 | 0.082 | 0.070 | 0.071 | 0.054 | 0.088 | 0.016 | 0.081 | 0.345 |

**Supplementary Table 3.** Weighted adjacency matrix from the COVID-19 outbreak period to the COVID-19 control period in the delayed dysfunction group.

|  | PHQ1 | PHQ2 | PHQ3 | PHQ4 | PHQ5 | PHQ6 | PHQ7 | PHQ8 | PHQ9 |
| --- | --- | --- | --- | --- | --- | --- | --- | --- | --- |
| PHQ1: Anhedonia | 0.036 | 0.000 | 0.000 | 0.000 | -0.022 | -0.008 | 0.016 | -0.021 | -0.045 |
| PHQ2: Depressed mood | -0.047 | 0.013 | -0.032 | -0.013 | -0.010 | -0.010 | -0.023 | 0.021 | 0.008 |
| PHQ3: Sleep | 0.000 | -0.014 | 0.099 | 0.000 | 0.020 | -0.028 | -0.025 | -0.047 | -0.017 |
| PHQ4: Lack of energy | 0.040 | 0.000 | 0.000 | 0.063 | 0.000 | -0.009 | 0.019 | -0.035 | -0.022 |
| PHQ5: Appetite | 0.000 | 0.000 | 0.000 | 0.000 | 0.093 | 0.000 | -0.020 | -0.010 | -0.016 |
| PHQ6: Guilt | 0.020 | 0.020 | -0.014 | 0.000 | -0.032 | 0.143 | -0.003 | -0.041 | 0.000 |
| PHQ7: Difficulty concentration | 0.001 | -0.015 | -0.012 | -0.007 | 0.000 | -0.006 | 0.086 | -0.017 | -0.039 |

**Supplementary Table 4.** Weighted adjacency matrix from the COVID-19 outbreak period to the COVID-19 control period in the recovery group.

|  | PHQ1 | PHQ2 | PHQ3 | PHQ4 | PHQ5 | PHQ6 | PHQ7 | PHQ8 | PHQ9 |
| --- | --- | --- | --- | --- | --- | --- | --- | --- | --- |
| PHQ1: Anhedonia | 0.041 | -0.026 | -0.001 | 0.000 | 0.000 | 0.000 | 0.000 | -0.001 | 0.000 |
| PHQ2: Depressed mood | -0.039 | 0.077 | 0.000 | 0.000 | 0.000 | -0.005 | -0.021 | 0.000 | 0.000 |
| PHQ3: Sleep | 0.010 | 0.016 | 0.098 | 0.027 | 0.000 | -0.025 | 0.005 | -0.008 | 0.000 |
| PHQ4: Lack of energy | -0.016 | -0.021 | 0.000 | 0.038 | 0.000 | 0.000 | 0.000 | -0.005 | 0.000 |
| PHQ5: Appetite | -0.017 | -0.013 | 0.000 | -0.006 | 0.082 | -0.003 | -0.014 | 0.000 | 0.000 |
| PHQ6: Guilt | -0.032 | -0.006 | 0.000 | -0.020 | -0.047 | 0.131 | -0.025 | -0.006 | 0.000 |
| PHQ7: Difficulty concentration | 0.000 | -0.020 | 0.000 | 0.000 | -0.017 | -0.007 | 0.053 | 0.000 | 0.000 |
| PHQ8: Motor | -0.020 | -0.014 | -0.017 | -0.017 | -0.014 | -0.066 | -0.007 | 0.028 | 0.000 |
| PHQ9: Suicidal ideation | -0.094 | -0.068 | -0.084 | -0.119 | -0.064 | -0.034 | -0.028 | 0.000 | 0.041 |

**Supplementary Table 5.** Weighted adjacency matrix from the COVID-19 outbreak period to the COVID-19 control period in the resistance group.

|  | PHQ1 | PHQ2 | PHQ3 | PHQ4 | PHQ5 | PHQ6 | PHQ7 | PHQ8 | PHQ9 |
| --- | --- | --- | --- | --- | --- | --- | --- | --- | --- |
| PHQ1: Anhedonia | 0.166 | 0.050 | 0.048 | 0.099 | 0.041 | 0.027 | 0.053 | 0.004 | 0.000 |
| PHQ2: Depressed mood | 0.035 | 0.158 | 0.043 | 0.052 | 0.023 | 0.040 | 0.017 | 0.014 | 0.000 |
| PHQ3: Sleep | 0.063 | 0.036 | 0.173 | 0.070 | 0.047 | 0.004 | 0.017 | 0.000 | 0.000 |
| PHQ4: Lack of energy | 0.105 | 0.074 | 0.074 | 0.169 | 0.076 | 0.026 | 0.043 | 0.007 | 0.000 |
| PHQ5: Appetite | 0.046 | 0.032 | 0.041 | 0.047 | 0.134 | 0.012 | 0.018 | 0.008 | 0.000 |
| PHQ6: Guilt | 0.068 | 0.064 | 0.001 | 0.039 | 0.010 | 0.198 | 0.028 | 0.010 | 0.005 |
| PHQ7: Difficulty concentration | 0.062 | 0.037 | 0.010 | 0.042 | 0.010 | 0.014 | 0.133 | 0.019 | 0.000 |
| PHQ8: Motor | 0.007 | 0.000 | 0.000 | -0.023 | 0.004 | -0.001 | 0.042 | 0.055 | 0.001 |
| PHQ9: Suicidal ideation | -0.059 | 0.000 | 0.002 | -0.022 | 0.000 | 0.046 | -0.022 | -0.003 | 0.051 |

**Supplementary Table 6.** The normality of each item of PHQ-8.

| **Symptoms** | | **Kolmogorov-Smirnov** | |
| --- | --- | --- | --- |
|  |  | **The COVID-19 outbreak period** | **The COVID-19 control period** |
| **PHQ1** | **Anhedonia** | 0.278*** | 0.301*** |
| **PHQ2** | **Depressed mood** | 0.362*** | 0.293*** |
| **PHQ3** | **Sleep** | 0.339*** | 0.314*** |
| **PHQ4** | **Little energy** | 0.326*** | 0.290*** |
| **PHQ5** | **Appetite** | 0.377*** | 0.332*** |
| **PHQ6** | **Guilt** | 0.413*** | 0.354*** |
| **PHQ7** | **Difficulty concentration** | 0.399*** | 0.350*** |
| **PHQ8** | **Motor** | 0.497*** | 0.444*** |
| **PHQ9** | **Suicidal ideation** | 0.530*** | 0.514*** |

The accuracy plots of four CLPNs show small-to-moderate confidence intervals around edge weights, suggesting good accuracy for the baseline to follow-up networks across four depressive trajectories (Supplementary Figure 1). Likewise, the case-drop bootstrapping results revealed that the rank order of in-EI and out-EI had moderate to strong stability across four CLPNs (Supplementary Figure 2). Specifically, CS coefficients of in-EI and out-EI were as follows: the chronic dysfunction group: CS _in-EI_ =0.693, CS _out-EI_ = 0.693; the delayed dysfunction group: CS _in-EI_ = 0.493, CS _out-EI_ = 0.336; the recovery group: CS _in-EI_ = 0.321, CS _out-EI_ = 0.679; the resistance group: CS _in-EI_ = 0.750, CS _out-EI_ = 0.750. In addition, the edge weights difference tests (Supplementary Figure 3) revealed that these edges were significantly stronger than most other edges, and centrality difference tests (Supplementary Figure 4-5) indicated that these symptoms displayed significantly higher OEI and IEI compared to other symptoms in the CLPNs.

**[Insert Supplementary Figure 1]**

**Supplementary Figure 1.** Bootstrapped 95% confidence intervals around each edge weight for the networks across four depressive trajectories. The gray area represents the 95% Confidence Intervals of edge weights. Red lines indicate the edge weight in the estimated sample network, while black lines indicate the values of each edge weight, ordered from the highest to the lowest value.

**[Insert Supplementary Figure 2]**

**Supplementary Figure 2**. Stability of centrality measures for the networks across four depressive trajectories. The y-axis represents the average correlations between the original network's centrality indices and the centrality indices from the networks that were re-estimated after excluding increasing percentages of cases.

**[Insert Supplementary Figure 3]**

**Supplementary Figure 3.** Edge weight difference tests for the networks across four depressive trajectories. Black boxes represent edges with a significant difference (*p* < 0.05), and gray boxes indicate edges without a significant difference from one another.

**[Insert Supplementary Figure 4]**

**Supplementary Figure 4.** Centrality difference tests of in expected inﬂuence for the network across four depressive trajectories. Black boxes indicate symptoms that significantly differ in centrality (*p* < 0.05), and gray boxes indicate symptoms whose centrality does not significantly differ.

**[Insert Supplementary Figure 5]**

**Supplementary Figure 5.** Centrality difference tests of out expected inﬂuence for the network across four depressive trajectories. Black boxes indicate symptoms that significantly differ in centrality (*p* < 0.05), and gray boxes indicate symptoms whose centrality does not significantly differ.

**Supplementary for the analytic script**

###Set and check of current working directory.

setwd("F:/CLPN_COVID-19_PHQ9_G")

getwd()

###Load package.

library(haven)

library(Matrix)

library(glmnet)

library(lavaan)

library(qgraph)

library(stargazer)

library(ggplot2)

library(bootnet)

library(vegan)

###Import .sav data.

Data <- read_sav("CLPN_Trajectories.sav")

###Node_Name.

Node_Name_Cov <- c("PHQ1", "PHQ2", "PHQ3", "PHQ4", "PHQ5", "PHQ6","PHQ7","PHQ8",

"PHQ9","Sex", "Age", "Severity of the COVID-19",

"Community infection",

"Relatives or friends being infected",

"Exposure media")

Node_Name <- c("PHQ1", "PHQ2", "PHQ3", "PHQ4", "PHQ5", "PHQ6","PHQ7","PHQ8",

"PHQ9")

#########Network structure: Chronic dysfunction#########

#########Network structure: Chronic dysfunction#########

Data_T1_T2_Chr_All <- subset(Data, Depressive_trajectories == '3')

Data_T1_T2_Chr_Raw <- Data_T1_T2_Chr_All[,5:28]

###Set up empty data frame to hold model variables only.

Data_T1_T2_Chr <- data.frame(Data_T1_T2_Chr_Raw)

###Re-number variables.

colnames(Data_T1_T2_Chr) <- c("PHQ1_T1", "PHQ2_T1", "PHQ3_T1",

"PHQ4_T1", "PHQ5_T1","PHQ6_T1",

"PHQ7_T1", "PHQ8_T1", "PHQ9_T1",

"PHQ1_T2", "PHQ2_T2", "PHQ3_T2",

"PHQ4_T2", "PHQ5_T2", "PHQ6_T2",

"PHQ7_T2", "PHQ8_T2", "PHQ9_T2",

"Sex", "Age", "Severity of the COVID-19",

"Community infection",

"Relatives or friends being infected",

"Exposure media")

###Set up the number of symptoms at each time point.

k <- 9

###Set up the number of covariate.

num_Cov <- 6

###Set up empty matrix of coefficients.

adjMat_Cov_T1_T2_Chr <- matrix(0, (k+num_Cov),(k+num_Cov))

###Estimate CLPN.

for (i in 1:k){

set.seed(1)

lassoreg_Cov_T1_T2_Chr <- cv.glmnet(data.matrix(Data_T1_T2_Chr[,c(1:k,(k*2+1):(k*2+num_Cov))]),

Data_T1_T2_Chr[,(k+i)], nfolds=10,

family="gaussian", alpha=1, standardize=TRUE)

lambda_Cov_T1_T2_Chr <- lassoreg_Cov_T1_T2_Chr$lambda.min

adjMat_Cov_T1_T2_Chr[(1:(k+num_Cov)),i] <- coef(lassoreg_Cov_T1_T2_Chr,

s=lambda_Cov_T1_T2_Chr,

exact=FALSE)[2:(num_Cov+k+1)]

}

###Remove covariates from adjacency matrix.

adjMat_Cov_T1_T2_Chr_R <- getWmat(adjMat_Cov_T1_T2_Chr, nNodes=k+num_Cov,

labels=Node_Name_Cov, diRested=T)

adjMat_T1_T2_Chr <- adjMat_Cov_T1_T2_Chr_R[1:k, 1:k]

stargazer(adjMat_T1_T2_Chr,type="html",out = "1.1_Adjacency_Matrix_T1_T2_Chr.doc")

###Save the autoregressive edges.

ARedges_T1_T2_Chr <- diag(adjMat_T1_T2_Chr)

stargazer(ARedges_T1_T2_Chr,type="html",out = "2.1_ARedges_T1_T2_Chr.doc")

###Identify the strongest edges (note: row=IV, col=DV).

res_T1_T2_Chr <- order(adjMat_T1_T2_Chr, decreasing = T)[seq_len(576)]

pos_T1_T2_Chr <- arrayInd(res_T1_T2_Chr, dim(adjMat_T1_T2_Chr), useNames = TRUE)

posWithLabs_T1_T2_Chr <- data.frame(nodeOut=pos_T1_T2_Chr[,1],

nodeIn=pos_T1_T2_Chr[,2],

value=adjMat_T1_T2_Chr[res_T1_T2_Chr])

write.table(posWithLabs_T1_T2_Chr, file="3.1_posWithLabs_T1_T2_Chr.csv",

col.names = FALSE)

### Save InExpectedInfluence.

Z_inEI_T1_T2_Chr <- centralityPlot(adjMat_T1_T2_Chr,include = c("InExpectedInfluence"))

Z_in_expectedTable_T1_T2_Chr <- Z_inEI_T1_T2_Chr$data[,c(3,5)]

write.table(Z_in_expectedTable_T1_T2_Chr, file="4.1_Z_in_expectedTable_T1_T2_Chr.csv",

col.names = FALSE)

### Save OutExpectedInfluence.

Z_outEI_T1_T2_Chr <- centralityPlot(adjMat_T1_T2_Chr,include = c("OutExpectedInfluence"))

Z_out_expectedTable_T1_T2_Chr <- Z_outEI_T1_T2_Chr$data[,c(3,5)]

write.table(Z_out_expectedTable_T1_T2_Chr, file="5.1_Z_out_expectedTable_T1_T2_Chr.csv",

col.names = FALSE)

#########Bootstrapping#########

#########Bootstrapping#########

CLPN_B_T1_T2_Chr <- function(Data_T1_T2_Chr) {

## create empty adjacency matrix

adjMat_Cov_T1_T2_Chr_B <- matrix(0, k+num_Cov, k+num_Cov)

## run CLPN loop to do series of nodewise regularized regressions

for (i in 1:9) {

# set.seed(1) # commented out so that it doesn't give the same answer every time when bootstrapping

lassoreg_Cov_T1_T2_Chr_B <- cv.glmnet(x=data.matrix(Data_T1_T2_Chr[,c(1:k,(k*2+1):(k*2+num_Cov))]),

y=Data_T1_T2_Chr[,(k+i)], nfolds=10, grouped=TRUE,

family="gaussian", alpha=1, standardize=TRUE)

lambda_Cov_T1_T2_Chr_B <- lassoreg_Cov_T1_T2_Chr_B$lambda.min

## paste coefficients into adjacency matrix

adjMat_Cov_T1_T2_Chr_B[1:(k+num_Cov),i] <- coef(lassoreg_Cov_T1_T2_Chr_B,

s=lambda_Cov_T1_T2_Chr_B,

exact=FALSE)[2:(k+num_Cov+1)]

}

## remove covariates from adjacency matrix

adjMat_T1_T2_Chr_B <- adjMat_Cov_T1_T2_Chr_B[1:k, 1:k]

return(adjMat_T1_T2_Chr_B)

}

###Estimate networks in bootnet (for bootstrapping).

set.seed(1)

net_T1_T2_Chr <- estimateNetwork(Data_T1_T2_Chr, fun=CLPN_B_T1_T2_Chr,

labels=Node_Name, directed=T)

set.seed(1)

nonParBoot_T1_T2_Chr <- bootnet(net_T1_T2_Chr, type="nonparametric",

nBoots=1000, directed=T,

statistics=c("edge","outExpectedInfluence",

"inExpectedInfluence"),

ncores=8)

###Plot Edge weight accuracy.

tiff("6.1_CLPN_T1_T2_Chr_Edge weight accuracy.tiff",width = 4800,

height = 4800,units = 'px',res=600,compression = "lzw")

plot(nonParBoot_T1_T2_Chr, labels=Node_Name, order = "sample")

# Close PDF device:

dev.off()

###Plot edge differences.

tiff("7.1_CLPN_T1_T2_Chr_Edge weight differences.tiff",width = 4800,

height = 4800,units = 'px',res=600,compression = "lzw")

plot(nonParBoot_T1_T2_Chr,

plot = "difference",

onlyNonZero = TRUE,

order = "sample")

# Close PDF device:

dev.off()

###Plot in-EI nodes differences.

tiff("8.1_CLPN_T1_T2_Chr_in-EI Nodes differences.tiff",width = 4800,

height = 4800,units = 'px',res=600,compression = "lzw")

plot(nonParBoot_T1_T2_Chr, "inExpectedInfluence",

plot = "difference",labels = TRUE,order="sample")

# Close PDF device:

dev.off()

###Plot out-EI nodes differences.

tiff("9.1_CLPN_T1_T2_Chr_out-EI Nodes differences.tiff",width = 4800,

height = 4800,units = 'px',res=600,compression = "lzw")

plot(nonParBoot_T1_T2_Chr, "outExpectedInfluence",

plot = "difference",labels = TRUE,order="sample")

# Close PDF device:

dev.off()

set.seed(1)

caseBoot_T1_T2_Chr <- bootnet(net_T1_T2_Chr, type="case", nBoots=1000,

directed=T, caseN = 50,

statistics=c( "outExpectedInfluence",

"inExpectedInfluence"), ncores=8)

###Compute CS-coefficients.

corStability(caseBoot_T1_T2_Chr, cor=.7)

### Plot centrality stability

tiff("10.1_CLPN_T1_T2_Chr_Centrality stability.tiff",width = 4800,

height = 4800,units = 'px',res=600,compression = "lzw")

plot(caseBoot_T1_T2_Chr,

statistics = c("outExpectedInfluence","inExpectedInfluence"))

# Close PDF device:

dev.off()

#########Network structure: Delayed dysfunction#########

#########Network structure: Delayed dysfunction#########

Data_T1_T2_Del_All <- subset(Data, Depressive_trajectories == '2')

Data_T1_T2_Del_Raw <- Data_T1_T2_Del_All[,5:28]

###Set up empty data frame to hold model variables only.

Data_T1_T2_Del <- data.frame(Data_T1_T2_Del_Raw)

###Re-number variables.

colnames(Data_T1_T2_Del) <- c("PHQ1_T1", "PHQ2_T1", "PHQ3_T1",

"PHQ4_T1", "PHQ5_T1","PHQ6_T1",

"PHQ7_T1", "PHQ8_T1", "PHQ9_T1",

"PHQ1_T2", "PHQ2_T2", "PHQ3_T2",

"PHQ4_T2", "PHQ5_T2", "PHQ6_T2",

"PHQ7_T2", "PHQ8_T2", "PHQ9_T2",

"Sex", "Age", "Severity of the COVID-19",

"Community infection",

"Relatives or friends being infected",

"Exposure media")

###Set up the number of symptoms at each time point.

k <- 9

###Set up the number of covariate.

num_Cov <- 6

###Set up empty matrix of coefficients.

adjMat_Cov_T1_T2_Del <- matrix(0, (k+num_Cov),(k+num_Cov))

###Estimate CLPN.

for (i in 1:k){

set.seed(1)

lassoreg_Cov_T1_T2_Del <- cv.glmnet(data.matrix(Data_T1_T2_Del[,c(1:k,(k*2+1):(k*2+num_Cov))]),

Data_T1_T2_Del[,(k+i)], nfolds=10,

family="gaussian", alpha=1, standardize=TRUE)

lambda_Cov_T1_T2_Del <- lassoreg_Cov_T1_T2_Del$lambda.min

adjMat_Cov_T1_T2_Del[(1:(k+num_Cov)),i] <- coef(lassoreg_Cov_T1_T2_Del,

s=lambda_Cov_T1_T2_Del,

exact=FALSE)[2:(num_Cov+k+1)]

}

###Remove covariates from adjacency matrix.

adjMat_Cov_T1_T2_Del_R <- getWmat(adjMat_Cov_T1_T2_Del, nNodes=k+num_Cov,

labels=Node_Name_Cov, diRested=T)

adjMat_T1_T2_Del <- adjMat_Cov_T1_T2_Del_R[1:k, 1:k]

stargazer(adjMat_T1_T2_Del,type="html",out = "1.2_Adjacency_Matrix_T1_T2_Del.doc")

###Save the autoregressive edges.

ARedges_T1_T2_Del <- diag(adjMat_T1_T2_Del)

stargazer(ARedges_T1_T2_Del,type="html",out = "2.2_ARedges_T1_T2_Del.doc")

###Identify the strongest edges (note: row=IV, col=DV).

res_T1_T2_Del <- order(adjMat_T1_T2_Del, decreasing = T)[seq_len(576)]

pos_T1_T2_Del <- arrayInd(res_T1_T2_Del, dim(adjMat_T1_T2_Del), useNames = TRUE)

posWithLabs_T1_T2_Del <- data.frame(nodeOut=pos_T1_T2_Del[,1],

nodeIn=pos_T1_T2_Del[,2],

value=adjMat_T1_T2_Del[res_T1_T2_Del])

write.table(posWithLabs_T1_T2_Del, file="3.2_posWithLabs_T1_T2_Del.csv",

col.names = FALSE)

### Save InExpectedInfluence.

Z_inEI_T1_T2_Del <- centralityPlot(adjMat_T1_T2_Del,include = c("InExpectedInfluence"))

Z_in_expectedTable_T1_T2_Del <- Z_inEI_T1_T2_Del$data[,c(3,5)]

write.table(Z_in_expectedTable_T1_T2_Del, file="4.2_Z_in_expectedTable_T1_T2_Del.csv",

col.names = FALSE)

### Save OutExpectedInfluence.

Z_outEI_T1_T2_Del <- centralityPlot(adjMat_T1_T2_Del,include = c("OutExpectedInfluence"))

Z_out_expectedTable_T1_T2_Del <- Z_outEI_T1_T2_Del$data[,c(3,5)]

write.table(Z_out_expectedTable_T1_T2_Del, file="5.2_Z_out_expectedTable_T1_T2_Del.csv",

col.names = FALSE)

#########Bootstrapping#########

#########Bootstrapping#########

CLPN_B_T1_T2_Del <- function(Data_T1_T2_Del) {

## create empty adjacency matrix

adjMat_Cov_T1_T2_Del_B <- matrix(0, k+num_Cov, k+num_Cov)

## run CLPN loop to do series of nodewise regularized regressions

for (i in 1:9) {

# set.seed(1) # commented out so that it doesn't give the same answer every time when bootstrapping

lassoreg_Cov_T1_T2_Del_B <- cv.glmnet(x=data.matrix(Data_T1_T2_Del[,c(1:k,(k*2+1):(k*2+num_Cov))]),

y=Data_T1_T2_Del[,(k+i)], nfolds=10, grouped=TRUE,

family="gaussian", alpha=1, standardize=TRUE)

lambda_Cov_T1_T2_Del_B <- lassoreg_Cov_T1_T2_Del_B$lambda.min

## paste coefficients into adjacency matrix

adjMat_Cov_T1_T2_Del_B[1:(k+num_Cov),i] <- coef(lassoreg_Cov_T1_T2_Del_B,

s=lambda_Cov_T1_T2_Del_B,

exact=FALSE)[2:(k+num_Cov+1)]

}

## remove covariates from adjacency matrix

adjMat_T1_T2_Del_B <- adjMat_Cov_T1_T2_Del_B[1:k, 1:k]

return(adjMat_T1_T2_Del_B)

}

###Estimate networks in bootnet (for bootstrapping).

set.seed(1)

net_T1_T2_Del <- estimateNetwork(Data_T1_T2_Del, fun=CLPN_B_T1_T2_Del,

labels=Node_Name, directed=T)

set.seed(1)

nonParBoot_T1_T2_Del <- bootnet(net_T1_T2_Del, type="nonparametric",

nBoots=1000, directed=T,

statistics=c("edge","outExpectedInfluence",

"inExpectedInfluence"),

ncores=8)

###Plot Edge weight accuracy.

tiff("6.2_CLPN_T1_T2_Del_Edge weight accuracy.tiff",width = 4800,

height = 4800,units = 'px',res=600,compression = "lzw")

plot(nonParBoot_T1_T2_Del, labels=Node_Name, order = "sample")

# Close PDF device:

dev.off()

###Plot edge differences.

tiff("7.2_CLPN_T1_T2_Del_Edge weight differences.tiff",width = 4800,

height = 4800,units = 'px',res=600,compression = "lzw")

plot(nonParBoot_T1_T2_Del,

plot = "difference",

onlyNonZero = TRUE,

order = "sample")

# Close PDF device:

dev.off()

###Plot in-EI nodes differences.

tiff("8.2_CLPN_T1_T2_Del_in-EI Nodes differences.tiff",width = 4800,

height = 4800,units = 'px',res=600,compression = "lzw")

plot(nonParBoot_T1_T2_Del, "inExpectedInfluence",

plot = "difference",labels = TRUE,order="sample")

# Close PDF device:

dev.off()

###Plot out-EI nodes differences.

tiff("9.2_CLPN_T1_T2_Del_out-EI Nodes differences.tiff",width = 4800,

height = 4800,units = 'px',res=600,compression = "lzw")

plot(nonParBoot_T1_T2_Del, "outExpectedInfluence",

plot = "difference",labels = TRUE,order="sample")

# Close PDF device:

dev.off()

set.seed(1)

caseBoot_T1_T2_Del <- bootnet(net_T1_T2_Del, type="case", nBoots=1000,

directed=T, caseN = 50,

statistics=c( "outExpectedInfluence",

"inExpectedInfluence"), ncores=8)

###Compute CS-coefficients.

corStability(caseBoot_T1_T2_Del, cor=.7)

### Plot centrality stability

tiff("10.2_CLPN_T1_T2_Del_Centrality stability.tiff",width = 4800,

height = 4800,units = 'px',res=600,compression = "lzw")

plot(caseBoot_T1_T2_Del,

statistics = c("outExpectedInfluence","inExpectedInfluence"))

# Close PDF device:

dev.off()

#########Network structure: Recover#########

#########Network structure: Recover#########

Data_T1_T2_Rec_All <- subset(Data, Depressive_trajectories == '1')

Data_T1_T2_Rec_Raw <- Data_T1_T2_Rec_All[,5:28]

###Set up empty data frame to hold model variables only.

Data_T1_T2_Rec <- data.frame(Data_T1_T2_Rec_Raw)

###Re-number variables.

colnames(Data_T1_T2_Rec) <- c("PHQ1_T1", "PHQ2_T1", "PHQ3_T1",

"PHQ4_T1", "PHQ5_T1","PHQ6_T1",

"PHQ7_T1", "PHQ8_T1", "PHQ9_T1",

"PHQ1_T2", "PHQ2_T2", "PHQ3_T2",

"PHQ4_T2", "PHQ5_T2", "PHQ6_T2",

"PHQ7_T2", "PHQ8_T2", "PHQ9_T2",

"Sex", "Age", "Severity of the COVID-19",

"Community infection",

"Relatives or friends being infected",

"Exposure media")

###Set up the number of symptoms at each time point.

k <- 9

###Set up the number of covariate.

num_Cov <- 6

###Set up empty matrix of coefficients.

adjMat_Cov_T1_T2_Rec <- matrix(0, (k+num_Cov),(k+num_Cov))

###Estimate CLPN.

for (i in 1:k){

set.seed(1)

lassoreg_Cov_T1_T2_Rec <- cv.glmnet(data.matrix(Data_T1_T2_Rec[,c(1:k,(k*2+1):(k*2+num_Cov))]),

Data_T1_T2_Rec[,(k+i)], nfolds=10,

family="gaussian", alpha=1, standardize=TRUE)

lambda_Cov_T1_T2_Rec <- lassoreg_Cov_T1_T2_Rec$lambda.min

adjMat_Cov_T1_T2_Rec[(1:(k+num_Cov)),i] <- coef(lassoreg_Cov_T1_T2_Rec,

s=lambda_Cov_T1_T2_Rec,

exact=FALSE)[2:(num_Cov+k+1)]

}

###Remove covariates from adjacency matrix.

adjMat_Cov_T1_T2_Rec_R <- getWmat(adjMat_Cov_T1_T2_Rec, nNodes=k+num_Cov,

labels=Node_Name_Cov, diRested=T)

adjMat_T1_T2_Rec <- adjMat_Cov_T1_T2_Rec_R[1:k, 1:k]

stargazer(adjMat_T1_T2_Rec,type="html",out = "1.3_Adjacency_Matrix_T1_T2_Rec.doc")

###Save the autoregressive edges.

ARedges_T1_T2_Rec <- diag(adjMat_T1_T2_Rec)

stargazer(ARedges_T1_T2_Rec,type="html",out = "2.3_ARedges_T1_T2_Rec.doc")

###Identify the strongest edges (note: row=IV, col=DV).

res_T1_T2_Rec <- order(adjMat_T1_T2_Rec, decreasing = T)[seq_len(576)]

pos_T1_T2_Rec <- arrayInd(res_T1_T2_Rec, dim(adjMat_T1_T2_Rec), useNames = TRUE)

posWithLabs_T1_T2_Rec <- data.frame(nodeOut=pos_T1_T2_Rec[,1],

nodeIn=pos_T1_T2_Rec[,2],

value=adjMat_T1_T2_Rec[res_T1_T2_Rec])

write.table(posWithLabs_T1_T2_Rec, file="3.3_posWithLabs_T1_T2_Rec.csv",

col.names = FALSE)

### Save InExpectedInfluence.

Z_inEI_T1_T2_Rec <- centralityPlot(adjMat_T1_T2_Rec,include = c("InExpectedInfluence"))

Z_in_expectedTable_T1_T2_Rec <- Z_inEI_T1_T2_Rec$data[,c(3,5)]

write.table(Z_in_expectedTable_T1_T2_Rec, file="4.3_Z_in_expectedTable_T1_T2_Rec.csv",

col.names = FALSE)

### Save OutExpectedInfluence.

Z_outEI_T1_T2_Rec <- centralityPlot(adjMat_T1_T2_Rec,include = c("OutExpectedInfluence"))

Z_out_expectedTable_T1_T2_Rec <- Z_outEI_T1_T2_Rec$data[,c(3,5)]

write.table(Z_out_expectedTable_T1_T2_Rec, file="5.3_Z_out_expectedTable_T1_T2_Rec.csv",

col.names = FALSE)

#########Bootstrapping#########

#########Bootstrapping#########

CLPN_B_T1_T2_Rec <- function(Data_T1_T2_Rec) {

## create empty adjacency matrix

adjMat_Cov_T1_T2_Rec_B <- matrix(0, k+num_Cov, k+num_Cov)

## run CLPN loop to do series of nodewise regularized regressions

for (i in 1:9) {

# set.seed(1) # commented out so that it doesn't give the same answer every time when bootstrapping

lassoreg_Cov_T1_T2_Rec_B <- cv.glmnet(x=data.matrix(Data_T1_T2_Rec[,c(1:k,(k*2+1):(k*2+num_Cov))]),

y=Data_T1_T2_Rec[,(k+i)], nfolds=10, grouped=TRUE,

family="gaussian", alpha=1, standardize=TRUE)

lambda_Cov_T1_T2_Rec_B <- lassoreg_Cov_T1_T2_Rec_B$lambda.min

## paste coefficients into adjacency matrix

adjMat_Cov_T1_T2_Rec_B[1:(k+num_Cov),i] <- coef(lassoreg_Cov_T1_T2_Rec_B,

s=lambda_Cov_T1_T2_Rec_B,

exact=FALSE)[2:(k+num_Cov+1)]

}

## remove covariates from adjacency matrix

adjMat_T1_T2_Rec_B <- adjMat_Cov_T1_T2_Rec_B[1:k, 1:k]

return(adjMat_T1_T2_Rec_B)

}

###Estimate networks in bootnet (for bootstrapping).

set.seed(1)

net_T1_T2_Rec <- estimateNetwork(Data_T1_T2_Rec, fun=CLPN_B_T1_T2_Rec,

labels=Node_Name, directed=T)

set.seed(1)

nonParBoot_T1_T2_Rec <- bootnet(net_T1_T2_Rec, type="nonparametric",

nBoots=1000, directed=T,

statistics=c("edge","outExpectedInfluence",

"inExpectedInfluence"),

ncores=8)

###Plot Edge weight accuracy.

tiff("6.3_CLPN_T1_T2_Rec_Edge weight accuracy.tiff",width = 4800,

height = 4800,units = 'px',res=600,compression = "lzw")

plot(nonParBoot_T1_T2_Rec, labels=Node_Name, order = "sample")

# Close PDF device:

dev.off()

###Plot edge differences.

tiff("7.3_CLPN_T1_T2_Rec_Edge weight differences.tiff",width = 4800,

height = 4800,units = 'px',res=600,compression = "lzw")

plot(nonParBoot_T1_T2_Rec,

plot = "difference",

onlyNonZero = TRUE,

order = "sample")

# Close PDF device:

dev.off()

###Plot in-EI nodes differences.

tiff("8.3_CLPN_T1_T2_Rec_in-EI Nodes differences.tiff",width = 4800,

height = 4800,units = 'px',res=600,compression = "lzw")

plot(nonParBoot_T1_T2_Rec, "inExpectedInfluence",

plot = "difference",labels = TRUE,order="sample")

# Close PDF device:

dev.off()

###Plot out-EI nodes differences.

tiff("9.3_CLPN_T1_T2_Rec_out-EI Nodes differences.tiff",width = 4800,

height = 4800,units = 'px',res=600,compression = "lzw")

plot(nonParBoot_T1_T2_Rec, "outExpectedInfluence",

plot = "difference",labels = TRUE,order="sample")

# Close PDF device:

dev.off()

set.seed(1)

caseBoot_T1_T2_Rec <- bootnet(net_T1_T2_Rec, type="case", nBoots=1000,

directed=T, caseN = 50,

statistics=c( "outExpectedInfluence",

"inExpectedInfluence"), ncores=8)

###Compute CS-coefficients.

corStability(caseBoot_T1_T2_Rec, cor=.7)

### Plot centrality stability

tiff("10.3_CLPN_T1_T2_Rec_Centrality stability.tiff",width = 4800,

height = 4800,units = 'px',res=600,compression = "lzw")

plot(caseBoot_T1_T2_Rec,

statistics = c("outExpectedInfluence","inExpectedInfluence"))

# Close PDF device:

dev.off()

#########Network structure: Resilience#########

#########Network structure: Resilience#########

Data_T1_T2_Res_All <- subset(Data, Depressive_trajectories == '0')

Data_T1_T2_Res_Raw <- Data_T1_T2_Res_All[,5:28]

###Set up empty data frame to hold model variables only.

Data_T1_T2_Res <- data.frame(Data_T1_T2_Res_Raw)

###Re-number variables.

colnames(Data_T1_T2_Res) <- c("PHQ1_T1", "PHQ2_T1", "PHQ3_T1",

"PHQ4_T1", "PHQ5_T1","PHQ6_T1",

"PHQ7_T1", "PHQ8_T1", "PHQ9_T1",

"PHQ1_T2", "PHQ2_T2", "PHQ3_T2",

"PHQ4_T2", "PHQ5_T2", "PHQ6_T2",

"PHQ7_T2", "PHQ8_T2", "PHQ9_T2",

"Sex", "Age", "Severity of the COVID-19",

"Community infection",

"Relatives or friends being infected",

"Exposure media")

###Set up the number of symptoms at each time point.

k <- 9

###Set up the number of covariate.

num_Cov <- 6

###Set up empty matrix of coefficients.

adjMat_Cov_T1_T2_Res <- matrix(0, (k+num_Cov),(k+num_Cov))

###Estimate CLPN.

for (i in 1:k){

set.seed(1)

lassoreg_Cov_T1_T2_Res <- cv.glmnet(data.matrix(Data_T1_T2_Res[,c(1:k,(k*2+1):(k*2+num_Cov))]),

Data_T1_T2_Res[,(k+i)], nfolds=10,

family="gaussian", alpha=1, standardize=TRUE)

lambda_Cov_T1_T2_Res <- lassoreg_Cov_T1_T2_Res$lambda.min

adjMat_Cov_T1_T2_Res[(1:(k+num_Cov)),i] <- coef(lassoreg_Cov_T1_T2_Res,

s=lambda_Cov_T1_T2_Res,

exact=FALSE)[2:(num_Cov+k+1)]

}

###Remove covariates from adjacency matrix.

adjMat_Cov_T1_T2_Res_R <- getWmat(adjMat_Cov_T1_T2_Res, nNodes=k+num_Cov,

labels=Node_Name_Cov, diRested=T)

adjMat_T1_T2_Res <- adjMat_Cov_T1_T2_Res_R[1:k, 1:k]

stargazer(adjMat_T1_T2_Res,type="html",out = "1.4_Adjacency_Matrix_T1_T2_Res.doc")

###Save the autoregressive edges.

ARedges_T1_T2_Res <- diag(adjMat_T1_T2_Res)

stargazer(ARedges_T1_T2_Res,type="html",out = "2.4_ARedges_T1_T2_Res.doc")

###Identify the strongest edges (note: row=IV, col=DV).

res_T1_T2_Res <- order(adjMat_T1_T2_Res, decreasing = T)[seq_len(576)]

pos_T1_T2_Res <- arrayInd(res_T1_T2_Res, dim(adjMat_T1_T2_Res), useNames = TRUE)

posWithLabs_T1_T2_Res <- data.frame(nodeOut=pos_T1_T2_Res[,1],

nodeIn=pos_T1_T2_Res[,2],

value=adjMat_T1_T2_Res[res_T1_T2_Res])

write.table(posWithLabs_T1_T2_Res, file="3.4_posWithLabs_T1_T2_Res.csv",

col.names = FALSE)

### Save InExpectedInfluence.

Z_inEI_T1_T2_Res <- centralityPlot(adjMat_T1_T2_Res,include = c("InExpectedInfluence"))

Z_in_expectedTable_T1_T2_Res <- Z_inEI_T1_T2_Res$data[,c(3,5)]

write.table(Z_in_expectedTable_T1_T2_Res, file="4.4_Z_in_expectedTable_T1_T2_Res.csv",

col.names = FALSE)

### Save OutExpectedInfluence.

Z_outEI_T1_T2_Res <- centralityPlot(adjMat_T1_T2_Res,include = c("OutExpectedInfluence"))

Z_out_expectedTable_T1_T2_Res <- Z_outEI_T1_T2_Res$data[,c(3,5)]

write.table(Z_out_expectedTable_T1_T2_Res, file="5.4_Z_out_expectedTable_T1_T2_Res.csv",

col.names = FALSE)

#########Bootstrapping#########

#########Bootstrapping#########

CLPN_B_T1_T2_Res <- function(Data_T1_T2_Res) {

## create empty adjacency matrix

adjMat_Cov_T1_T2_Res_B <- matrix(0, k+num_Cov, k+num_Cov)

## run CLPN loop to do series of nodewise regularized regressions

for (i in 1:9) {

# set.seed(1) # commented out so that it doesn't give the same answer every time when bootstrapping

lassoreg_Cov_T1_T2_Res_B <- cv.glmnet(x=data.matrix(Data_T1_T2_Res[,c(1:k,(k*2+1):(k*2+num_Cov))]),

y=Data_T1_T2_Res[,(k+i)], nfolds=10, grouped=TRUE,

family="gaussian", alpha=1, standardize=TRUE)

lambda_Cov_T1_T2_Res_B <- lassoreg_Cov_T1_T2_Res_B$lambda.min

## paste coefficients into adjacency matrix

adjMat_Cov_T1_T2_Res_B[1:(k+num_Cov),i] <- coef(lassoreg_Cov_T1_T2_Res_B,

s=lambda_Cov_T1_T2_Res_B,

exact=FALSE)[2:(k+num_Cov+1)]

}

## remove covariates from adjacency matrix

adjMat_T1_T2_Res_B <- adjMat_Cov_T1_T2_Res_B[1:k, 1:k]

return(adjMat_T1_T2_Res_B)

}

###Estimate networks in bootnet (for bootstrapping).

set.seed(1)

net_T1_T2_Res <- estimateNetwork(Data_T1_T2_Res, fun=CLPN_B_T1_T2_Res,

labels=Node_Name, directed=T)

set.seed(1)

nonParBoot_T1_T2_Res <- bootnet(net_T1_T2_Res, type="nonparametric",

nBoots=1000, directed=T,

statistics=c("edge","outExpectedInfluence",

"inExpectedInfluence"),

ncores=8)

###Plot Edge weight accuracy.

tiff("6.4_CLPN_T1_T2_Res_Edge weight accuracy.tiff",width = 4800,

height = 4800,units = 'px',res=600,compression = "lzw")

plot(nonParBoot_T1_T2_Res, labels=Node_Name, order = "sample")

# Close PDF device:

dev.off()

###Plot edge differences.

tiff("7.4_CLPN_T1_T2_Res_Edge weight differences.tiff",width = 4800,

height = 4800,units = 'px',res=600,compression = "lzw")

plot(nonParBoot_T1_T2_Res,

plot = "difference",

onlyNonZero = TRUE,

order = "sample")

# Close PDF device:

dev.off()

###Plot in-EI nodes differences.

tiff("8.4_CLPN_T1_T2_Res_in-EI Nodes differences.tiff",width = 4800,

height = 4800,units = 'px',res=600,compression = "lzw")

plot(nonParBoot_T1_T2_Res, "inExpectedInfluence",

plot = "difference",labels = TRUE,order="sample")

# Close PDF device:

dev.off()

###Plot out-EI nodes differences.

tiff("9.4_CLPN_T1_T2_Res_out-EI Nodes differences.tiff",width = 4800,

height = 4800,units = 'px',res=600,compression = "lzw")

plot(nonParBoot_T1_T2_Res, "outExpectedInfluence",

plot = "difference",labels = TRUE,order="sample")

# Close PDF device:

dev.off()

set.seed(1)

caseBoot_T1_T2_Res <- bootnet(net_T1_T2_Res, type="case", nBoots=1000,

directed=T, caseN = 50,

statistics=c( "outExpectedInfluence",

"inExpectedInfluence"), ncores=8)

###Compute CS-coefficients.

corStability(caseBoot_T1_T2_Res, cor=.7)

### Plot centrality stability

tiff("10.4_CLPN_T1_T2_Res_Centrality stability.tiff",width = 4800,

height = 4800,units = 'px',res=600,compression = "lzw")

plot(caseBoot_T1_T2_Res,

statistics = c("outExpectedInfluence","inExpectedInfluence"))

# Close PDF device:

dev.off()

###Plot CLPN.

maxEdge <- max(c(adjMat_T1_T2_Chr,adjMat_T1_T2_Del,adjMat_T1_T2_Rec,adjMat_T1_T2_Res))

layoutwoAR <- averageLayout(adjMat_T1_T2_Chr,adjMat_T1_T2_Del,adjMat_T1_T2_Rec,adjMat_T1_T2_Res)

tiff("11.1_CLPN_Chr_T1_T2.tiff", width = 6000, height = 5800,

units = 'px', res=600, compression = "lzw")

plot_T1_T2 <- qgraph(adjMat_T1_T2_Chr, labels=Node_Name,layout=layoutwoAR,

legend=F,maximum=maxEdge,

label.prop=0.9,color=c("#E69F00"),

asize=3,vsize=9, label.cex=1,threshold=0.025,

GLratio=4,node.width=1,node.height=1,cut=0)

dev.off()

tiff("11.2_CLPN_Del_T1_T2.tiff", width = 6000, height = 5800,

units = 'px', res=600, compression = "lzw")

plot_T1_T2 <- qgraph(adjMat_T1_T2_Del, labels=Node_Name,layout=layoutwoAR,

legend=F,maximum=maxEdge,

label.prop=0.9,color=c("#E69F00"),

asize=3,vsize=9, label.cex=1,threshold=0.025,

GLratio=4,node.width=1,node.height=1,cut=0)

dev.off()

tiff("11.3_CLPN_Rec_T1_T2.tiff", width = 6000, height = 5800,

units = 'px', res=600, compression = "lzw")

plot_T1_T2 <- qgraph(adjMat_T1_T2_Rec, labels=Node_Name,layout=layoutwoAR,

legend=F,maximum=maxEdge,

label.prop=0.9,color=c("#E69F00"),

asize=3,vsize=9, label.cex=1,threshold=0.025,

GLratio=4,node.width=1,node.height=1,cut=0)

dev.off()

tiff("11.4_CLPN_Res_T1_T2.tiff", width = 6000, height = 5800,

units = 'px', res=600, compression = "lzw")

plot_T1_T2 <- qgraph(adjMat_T1_T2_Res, labels=Node_Name,layout=layoutwoAR,

legend=F,maximum=maxEdge,

label.prop=0.9,color=c("#E69F00"),

asize=3,vsize=9, label.cex=1,threshold=0.025,

GLratio=4,node.width=1,node.height=1,cut=0)

dev.off()

###Correlation between overall and sub-groups.

mantel(adjMat_T1_T2_Chr,adjMat_T1_T2_Del,method='spearman')

mantel(adjMat_T1_T2_Chr,adjMat_T1_T2_Rec,method='spearman')

mantel(adjMat_T1_T2_Chr,adjMat_T1_T2_Res,method='spearman')

mantel(adjMat_T1_T2_Del,adjMat_T1_T2_Rec,method='spearman')

mantel(adjMat_T1_T2_Del,adjMat_T1_T2_Res,method='spearman')

mantel(adjMat_T1_T2_Rec,adjMat_T1_T2_Res,method='spearman')
