# Supplementary figures and images for: Temporal Network of Depressive Symptoms across College Students with Distinct Depressive Trajectories during the COVID-19 Pandemic
Source: Depress Anxiety. 2023 Jul 15;2023:8469620. doi: 10.1155/2023/8469620 (PMC11921855; doi:10.1155/2023/8469620)

## Slide 1
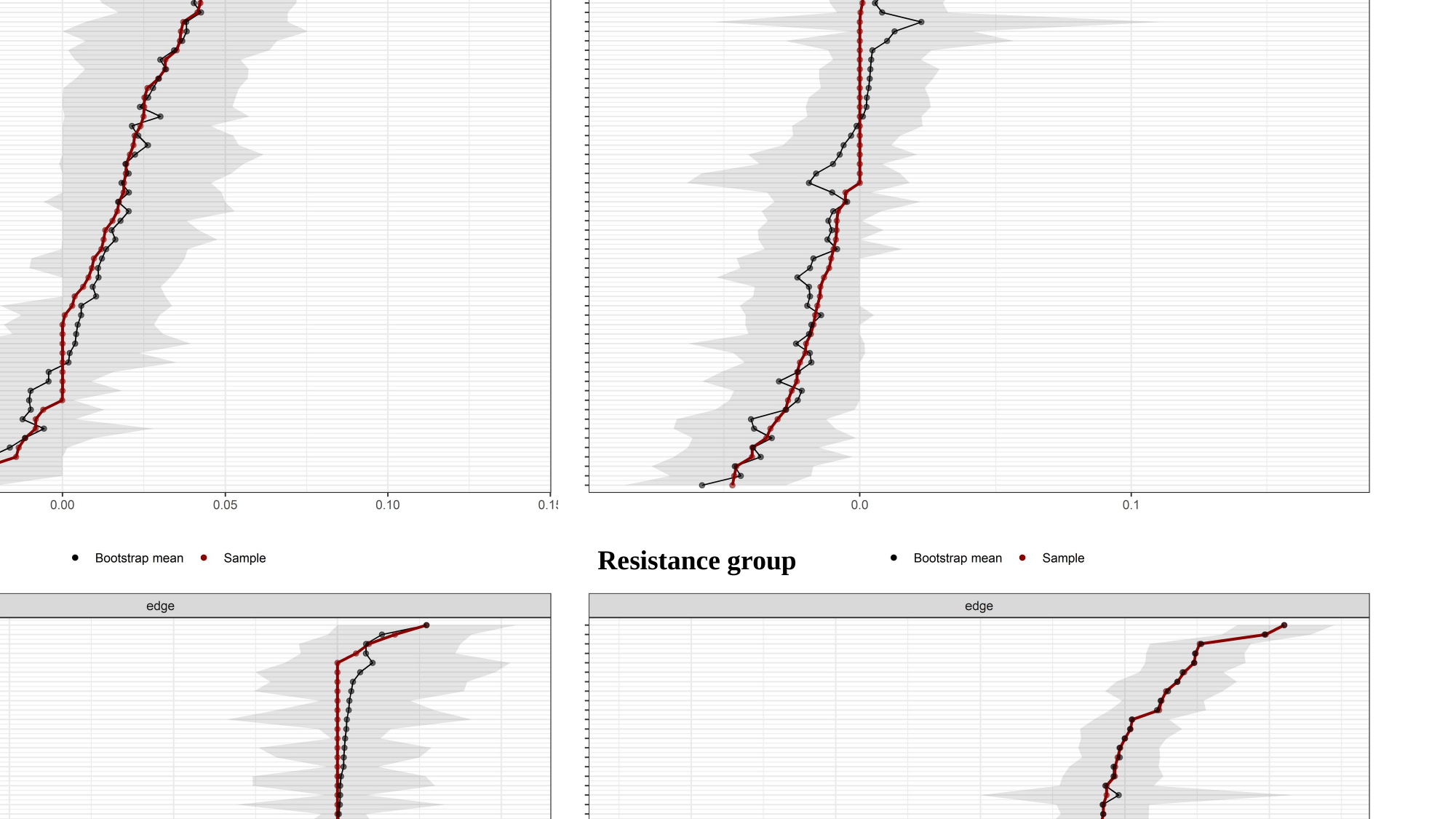

Chronic dysfunction group Delayed dysfunction group
Recovery group Resistance group

Supplement: Supplementary Materials — Detailed sample information is shown in Supplementary Table 1. All edge weights are presented in Supplementary Tables 2–5. Supplementary Figures 1–5 provide the results for accuracy (Supplementary Figure 1) and stability (Supplementary Figure 1) of the network, the edge weight difference tests (Supplementary Figure 3), and centrality difference tests (Supplementary Figures 4 and 5). [file 8469620.f1.zip › Supplementary Figure 1.pptx]

## Slide 1
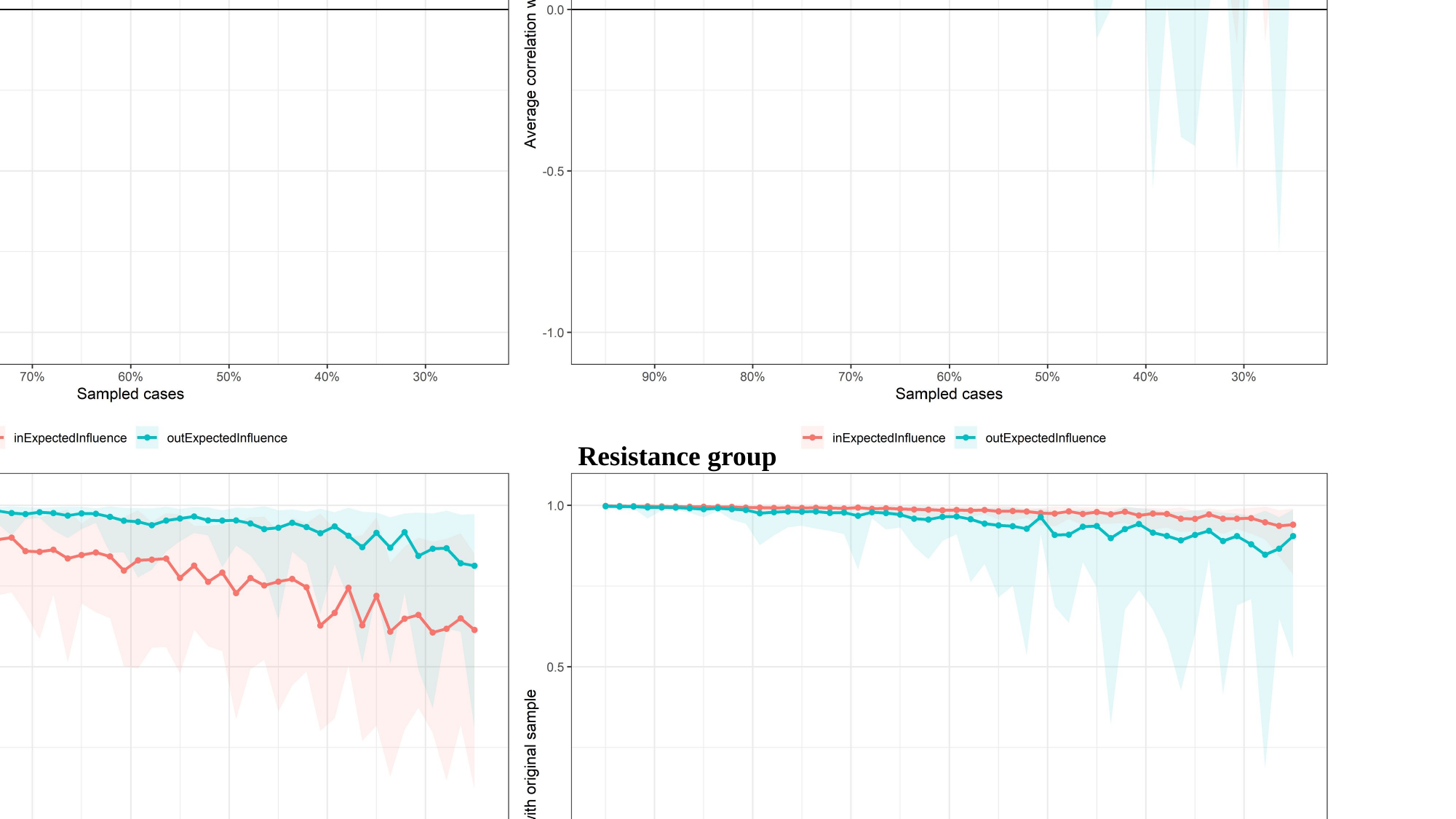

Chronic dysfunction group Delayed dysfunction group
Recovery group Resistance group

Supplement: Supplementary Materials — Detailed sample information is shown in Supplementary Table 1. All edge weights are presented in Supplementary Tables 2–5. Supplementary Figures 1–5 provide the results for accuracy (Supplementary Figure 1) and stability (Supplementary Figure 1) of the network, the edge weight difference tests (Supplementary Figure 3), and centrality difference tests (Supplementary Figures 4 and 5). [file 8469620.f1.zip › Supplementary Figure 2.pptx]

## Slide 1
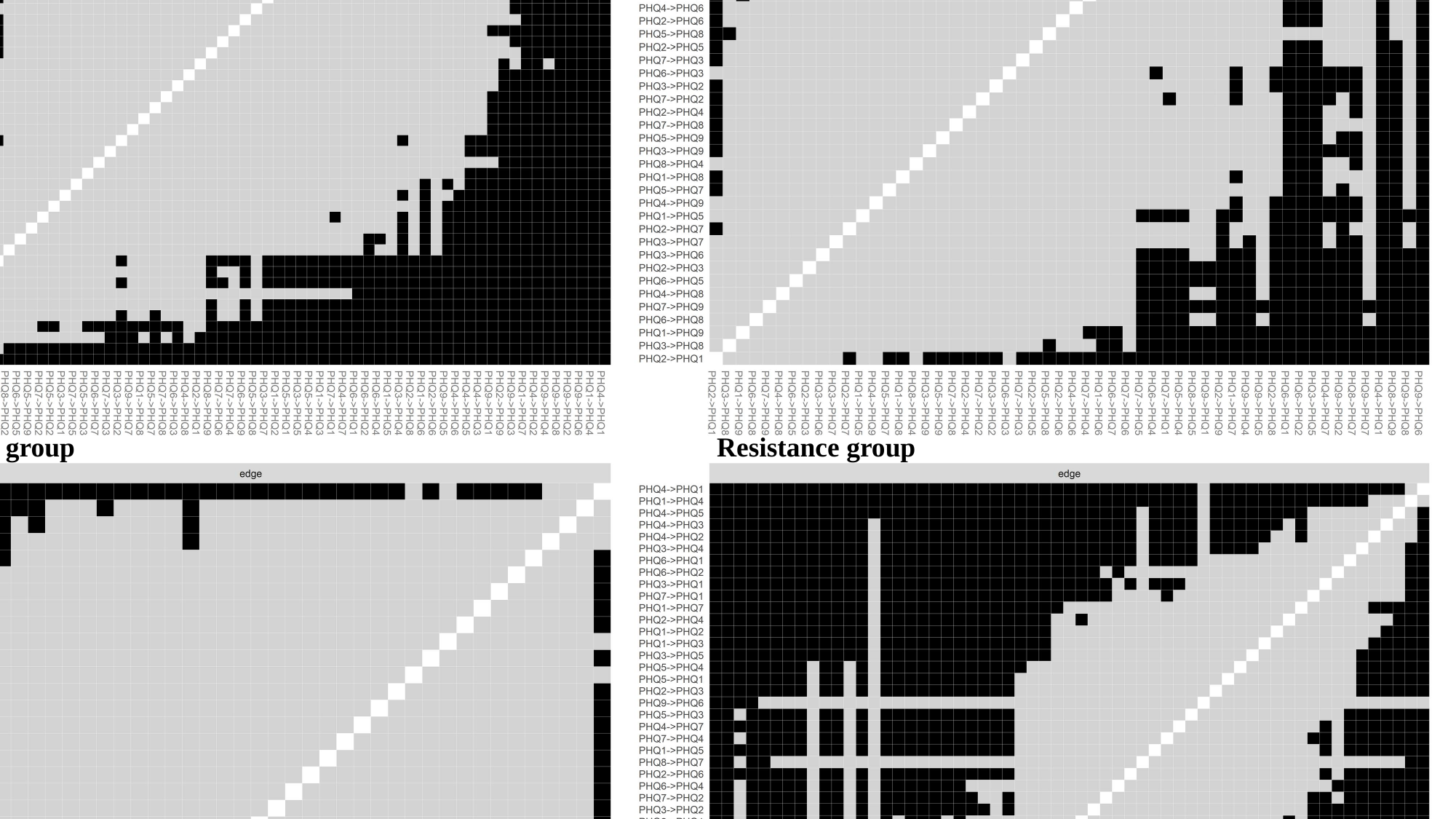

Chronic dysfunction group Delayed dysfunction group
Recovery group Resistance group

Supplement: Supplementary Materials — Detailed sample information is shown in Supplementary Table 1. All edge weights are presented in Supplementary Tables 2–5. Supplementary Figures 1–5 provide the results for accuracy (Supplementary Figure 1) and stability (Supplementary Figure 1) of the network, the edge weight difference tests (Supplementary Figure 3), and centrality difference tests (Supplementary Figures 4 and 5). [file 8469620.f1.zip › Supplementary Figure 3.pptx]

## Slide 1
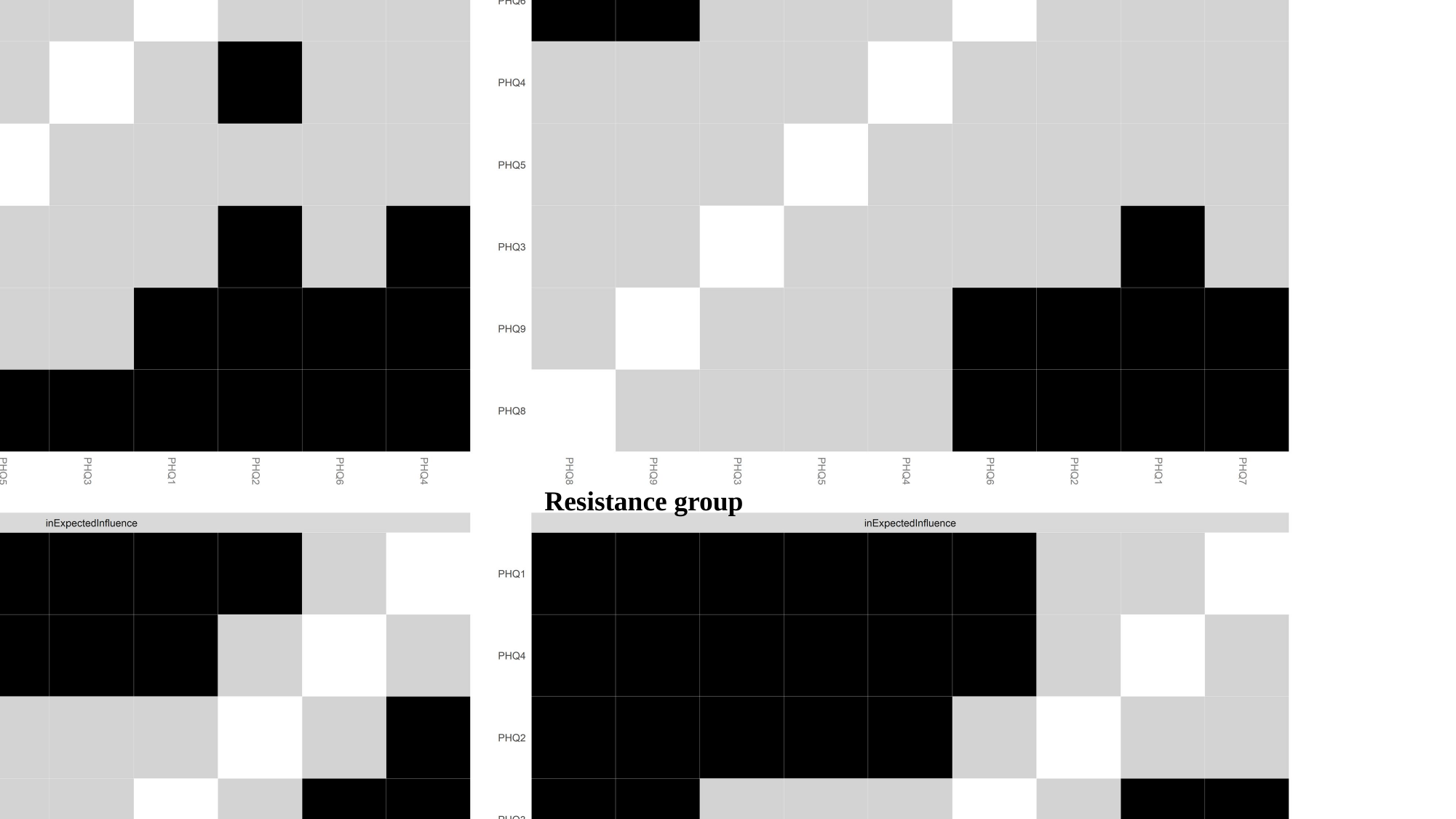

Chronic dysfunction group Delayed dysfunction group
Recovery group Resistance group

Supplement: Supplementary Materials — Detailed sample information is shown in Supplementary Table 1. All edge weights are presented in Supplementary Tables 2–5. Supplementary Figures 1–5 provide the results for accuracy (Supplementary Figure 1) and stability (Supplementary Figure 1) of the network, the edge weight difference tests (Supplementary Figure 3), and centrality difference tests (Supplementary Figures 4 and 5). [file 8469620.f1.zip › Supplementary Figure 4.pptx]

## Slide 1
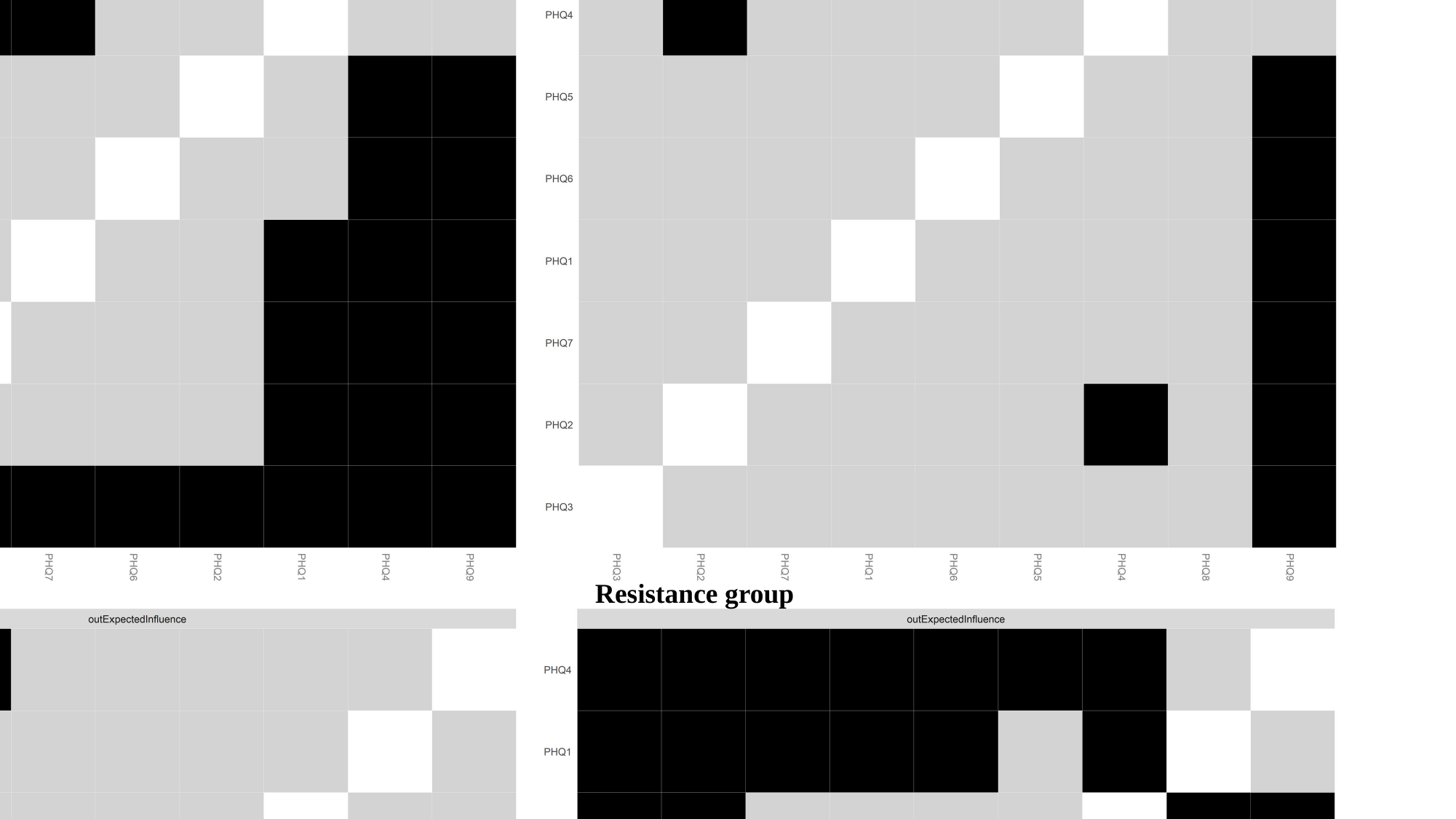

Chronic dysfunction group Delayed dysfunction group
Recovery group Resistance group

Supplement: Supplementary Materials — Detailed sample information is shown in Supplementary Table 1. All edge weights are presented in Supplementary Tables 2–5. Supplementary Figures 1–5 provide the results for accuracy (Supplementary Figure 1) and stability (Supplementary Figure 1) of the network, the edge weight difference tests (Supplementary Figure 3), and centrality difference tests (Supplementary Figures 4 and 5). [file 8469620.f1.zip › Supplementary Figure 5.pptx]
